# The State of Infectious Diseases Clinical Trials: A Systematic Review of ClinicalTrials.gov

**DOI:** 10.1371/journal.pone.0077086

**Published:** 2013-10-16

**Authors:** Neela D. Goswami, Christopher D. Pfeiffer, John R. Horton, Karen Chiswell, Asba Tasneem, Ephraim L. Tsalik

**Affiliations:** 1 Division of Infectious Diseases and International Health, Department of Medicine, Duke University School of Medicine, Durham, North Carolina, United States of America; 2 Department of Hospital and Specialty Medicine, Portland VA Medical Center, Portland, Oregon, United States of America; 3 Division of Infectious Diseases, Oregon Health and Science University, Portland, Oregon, United States of America; 4 Duke Clinical Research Institute, Durham, North Carolina, United States of America; 5 Emergency Medicine Service, Durham VA Medical Center, Durham, North Carolina, United States of America; University Medical Center Göttingen, Germany

## Abstract

**Background:**

There is a paucity of clinical trials informing specific questions faced by infectious diseases (ID) specialists. The ClinicalTrials.gov registry offers an opportunity to evaluate the ID clinical trials portfolio.

**Methods:**

We examined 40,970 interventional trials registered with ClinicalTrials.gov from 2007–2010, focusing on study conditions and interventions to identify ID-related trials. Relevance to ID was manually confirmed for each programmatically identified trial, yielding 3570 ID trials and 37,400 non-ID trials for analysis.

**Results:**

The number of ID trials was similar to the number of trials identified as belonging to cardiovascular medicine (n = 3437) or mental health (n = 3695) specialties. Slightly over half of ID trials were treatment-oriented trials (53%, vs. 77% for non-ID trials) followed by prevention (38%, vs. 8% in non-ID trials). ID trials tended to be larger than those of other specialties, with a median enrollment of 125 subjects (interquartile range [IQR], 45–400) vs. 60 (IQR, 30–160) for non-ID trials. Most ID studies are randomized (73%) but nonblinded (56%). Industry was the funding source in 51% of ID trials vs. 10% that were primarily NIH-funded. HIV-AIDS trials constitute the largest subset of ID trials (n = 815 [23%]), followed by influenza vaccine (n = 375 [11%]), and hepatitis C (n = 339 [9%]) trials. Relative to U.S. and global mortality rates, HIV-AIDS and hepatitis C virus trials are over-represented, whereas lower respiratory tract infection trials are under-represented in this large sample of ID clinical trials.

**Conclusions:**

This work is the first to characterize ID clinical trials registered in ClinicalTrials.gov, providing a framework to discuss prioritization, methodology, and policy.

## Introduction

In the context of modern, evidence-based prevention and treatment of infectious diseases, clinical trials provide information about the accuracy of molecular or microbiological diagnostics, the prognosis of infectious syndromes, and the potential risks and benefits of anti-infective regimens. However, there is a paucity of clinical trials informing the specific questions faced by infectious disease (ID) specialists. More than 50% of the recommendations contained in the practice guidelines published by the Infectious Diseases Society of America (IDSA) are based solely upon expert opinion; less than a quarter are based on evidence from randomized controlled trials (RCTs) [1], [2]. There is also increasing concern about the role of the medical products industry in the design and conduct of research, and in formulating practice guidelines [3]. But despite these concerns, the current spectrum of ID clinical trials has largely gone without systematic scrutiny regarding patterns of topical focus, geographical distribution, and levels of industry involvement.

ClinicalTrials.gov, a U.S. Food and Drug Administration (FDA)-initiated registry of more than 100,000 trials from 174 countries, provides a unique opportunity to take a “snapshot” of ID trials in terms of content and sponsorship. In September of 2007, registration of trials of drugs, biologics, and devices with ClinicalTrials.gov became a legal requirement for a large segment of clinical research conducted under U.S. jurisdiction; in addition, many peer-reviewed journals require registration with ClinicalTrials.gov or a comparable registry as a condition of publication [4]. Although clinical trials as a whole have been summarized from this registry [5], [6], ID trials have not been described, in part because they had not been prospectively designated as infection-related trials.

Recently, the Clinical Trials Transformation Initiative (CTTI) transformed the ClinicalTrials.gov database into a searchable relational dataset of registry content and regrouped studies into clinical specialties, thereby allowing targeted analysis. In this cross-sectional study, we aimed to characterize the scope and nature of ID clinical trials in this registry through a systematic analysis of characteristics of the registered trials, including trial methodology, geographic distribution, and funding source. We also evaluated the alignment between current clinical research priorities and the infections that cause the highest morbidity and mortality in the United States and around the world.

## Materials and Methods

We performed a systematic analysis of characteristics of infectious disease trials registered with ClinicalTrials.gov from October 1, 2007 to September 27, 2010. We chose ClinicalTrials.gov for our analysis over other registries because of its large size, international field of studies, publically available data element definitions and design details, inclusion of a full range of clinical conditions, broad group of trial sponsors [7], and its regulatory mandate [8], and also because ClinicalTrials.gov is the largest registry to allow bulk download of its entire dataset. The methods used by ClinicalTrials.gov to register clinical trials have been described previously [7], [9], [10]. Briefly, trial sponsors and investigators from around the world can enter trial data through a Web-based data entry system. The sample we examined in the present study includes trials that were registered to meet legal obligations such as from the FDA Amendments Act of 2007, as well as those registered to comply with requirements for peer-reviewed publication by the International Committee of Medical Journal Editors [4].

### Creation of the ID Study Dataset

**Figure 1:**
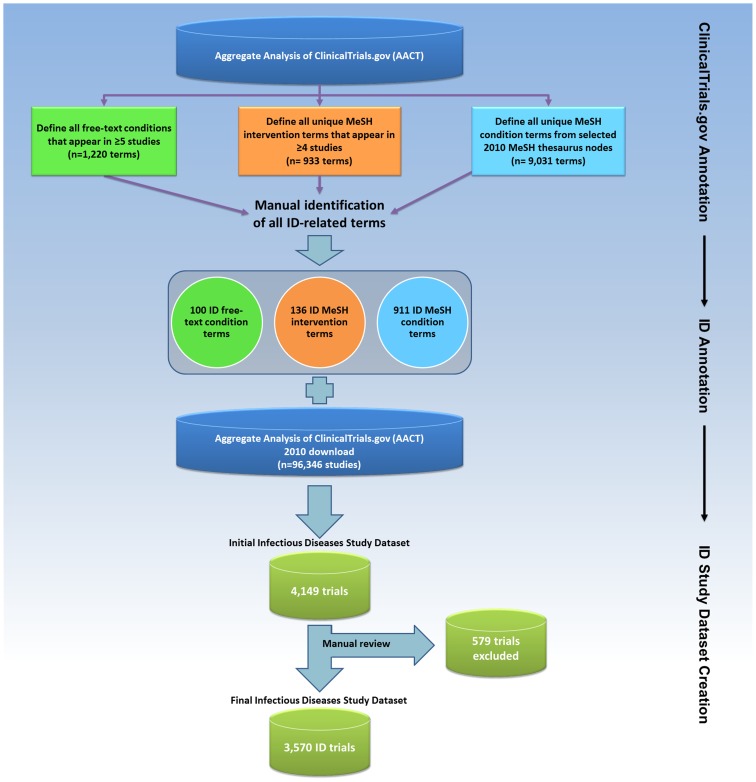
Flow diagram depicting the derivation of the Infectious Diseases Trials Dataset.

We downloaded a dataset comprising all 96,346 clinical trials registered with ClinicalTrials.gov from its inception through September 27, 2010. We focused on trials registered on or after October 2007, as this date corresponded with the beginning of the U.S. legal requirement [8] for registering certain trials of drugs, biologics, and devices. We next designed and implemented a relational database (Oracle RDBMS, version 11.1 g [Oracle Corporation, Redwood Shores, California, USA]) to analyze the aggregate data. The resulting Aggregate Analysis of ClincalTrials.gov (AACT) database is publicly available, along with data definitions and comprehensive data dictionaries, at the CTTI website [11]. We focused on interventional trials, which were identified based on the *study type* field included in the ClinicalTrials.gov registry. This field offers 4 options: Interventional, Observational, Expanded Access, and Not Applicable [11]. We used *study type* field as a filter, which resulted in 40,970 interventional trials registered during the 3-year period from October 2007 to September 2010 (Figure 1).

Next, we regrouped studies by clinical specialties within this derivative database [12]. In order to identify trials that were potentially relevant to ID, we focused on 2 trial characteristics: *condition* and *intervention*, which were defined either by data submitters or linked to Medical Subject Heading (MeSH) terms generated by a National Library of Medicine (NLM) algorithm based on the 2010 MeSH thesaurus [12]. A manual review of 9031 unique condition-related MeSH terms identified 911 (10%) terms relevant to ID. Not all conditions could be linked to MeSH terms; therefore, free-text condition terms appearing in 5 or more studies were also annotated for relevance to ID. Using this approach, out of 1220 unique, frequently occurring free-text condition terms, we identified 100 (8%) ID-related terms.

The second trial characteristic used to identify ID-relevant studies was the *intervention* term. We focused only on intervention terms that appeared in 4 or more studies and reviewed these for relevance to ID. This process identified 136 ID-related intervention MeSH terms out of 933 reviewed (15%). The results of algorithmic classifications were validated by comparison with classifications based on manual review [12]. All annotations were performed by 3 ID physicians (N.D.G., C.D.P., and E.L.T.). The identified terms relevant to ID are listed in Appendix S1.

Using a computer-based search, we identified 4149 studies with at least 1 ID-relevant term in the NLM-generated MeSH “condition” field, the submitted free-text “condition” field, or the submitted intervention name field. The authors (N.D.G., C.D.P., and E.L.T.) then manually reviewed each study to exclude non-ID studies and assign each study to an ID subcategory. Trials were included if they focused on a communicable disease. Trials evaluating non-communicable diseases were included only if the study hypothesis was that microbes played a role in the disease under study (e.g., probiotics to prevent hepatic encephalopathy). Studies that examined ID complications were included only if the intervention focused on the infection itself (e.g., post-herpetic neuralgia that included antiviral therapy).

Additional details are available in supplemental material (Checklist S1 and Diagram S1).

### Subcategorization of the ID Study Dataset

**Figure 2:**
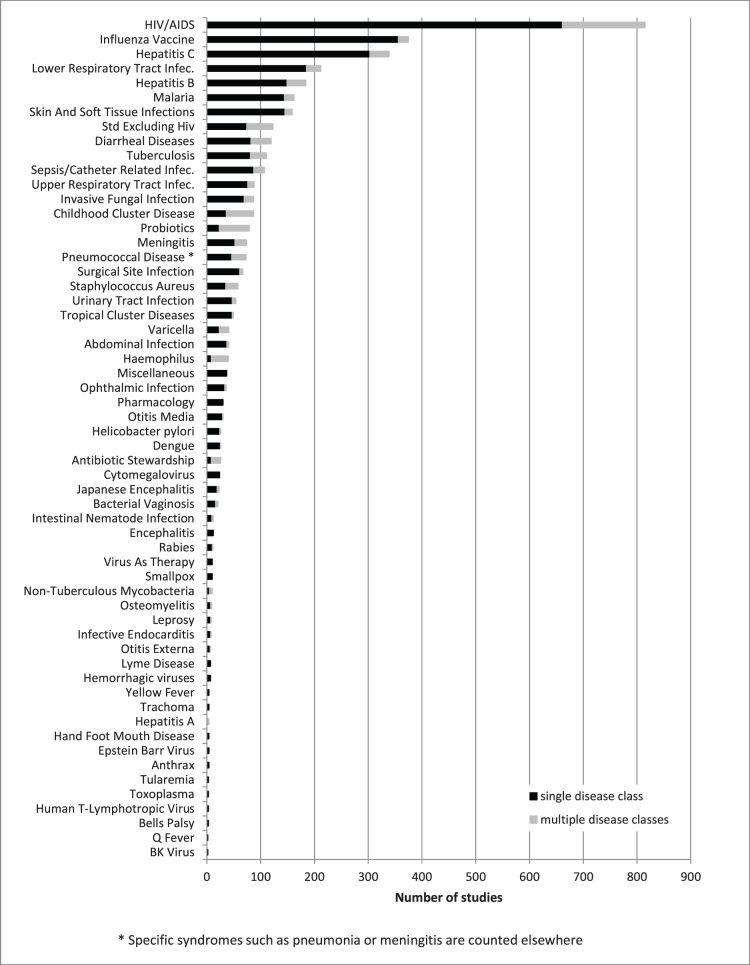
Number of trials in each of the 58 infectious disease (ID) subcategories comprising the ID Trials Dataset. The grey bar represents the trials assigned to more than 1 subcategory.

After defining the ID trials dataset, we subcategorized trials based on study title and description. When possible, subcategories were defined based on World Health Organization (WHO) cause-of-death groupings [13]. “Maternal Conditions” and “Perinatal Conditions” were excluded, because these categories include both communicable and non-communicable conditions. In addition to these 18 WHO-defined categories, 40 additional subcategories (for a total of 58) were defined such that each trial was assigned to at least 1 subcategory (Figure 2). Trials that fit equally well into multiple subcategories were assigned to more than 1 category. The percentage of all ID-related mortality and ID-related disability-adjusted life years (DALY) attributable to the following conditions was calculated from the WHO Global Burden of Disease [13]–[15]: HIV-AIDS, hepatitis C, lower respiratory tract infection (LRTI), hepatitis B, malaria, diarrheal diseases, sexually transmitted diseases (STD) excluding HIV, tuberculosis, childhood cluster diseases (pertussis, poliomyelitis, diphtheria, measles, and tetanus), and meningitis.

### Analytical Methods

SAS software, version 9.2 (SAS Institute, Cary, NC, USA) was used to calculate frequencies and percentages for categorical trial characteristics and median and interquartile ranges (IQRs) for continuous characteristics. A chi-squared test was used to evaluate whether trials that were focused on prevention or vaccines were more likely to restrict enrollment to children than non-prevention or non-vaccine trials, respectively. Disease prevalence and disease-specific DALYs were derived from the WHO 2004 Global Burden of Disease report, whereas disease-specific mortality was derived from its 2008 update [13]–[15]. Enrollment (number of trial participants) was reported for each trial. For active trials that had not completed recruitment, the anticipated enrollment was recorded. For trials that had completed or terminated recruitment, the value was updated to record actual enrollment. Summary statistics were calculated by pooling across active and completed trials. Because ClinicalTrials.gov does not require definition of the funding source for any given study, we derived the probable funding source based on the *lead sponsor* and *collaborator* fields [6]. The lead sponsor is the primary organization that oversees study implementation and is responsible for conducting data analysis [11]; available categories include “Industry”, “NIH”, “U.S. federal (excluding NIH)”, or “Other”. *Collaborators* are other organizations that provide additional support including funding, design, implementation, data analysis, or reporting. A trial was considered to be “industry-funded” if the lead sponsor was from industry, or if the NIH was neither a lead sponsor nor collaborator and at least 1 collaborator was from industry. An “NIH-funded” study required the NIH to be either a lead sponsor or a collaborator, and no industry as lead sponsor. “Other” was used to describe studies for which the lead sponsor and collaborator fields were non-missing and did not meet criteria for either “industry-funded” or “NIH-funded”. To further define this lead sponsor group, we undertook a manual review to assign trial sponsors to “Non-U.S. Government”, “Academic/Hospital”, or “Consortium”. Location of study facilities (sites) was defined by the study sponsor and could include more than 1 location for multisite studies. This information was used to define the regional distribution within the ID trials dataset.

Countries were grouped into 11 global regions (http://www.clinicaltrials.gov/ct2/search/browse?brwse=locn_cat). For each of 7 disease conditions (LRTI, Diarrheal diseases, HIV/AIDS, Tuberculosis, Malaria, Hepatitis B, and Hepatitis C), regions were ranked by the number of disease-specific studies with sites in a region, and by the total number of disease-specific deaths in a region [15]. These 2 rankings were compared graphically to examine the geographic distribution of trials in a specific disease relative to the geographic variation in mortality for that disease.

### Funding

This work was supported by a grant from the FDA awarded to Duke University. The study sponsor had no role in the design or performance of this study, or in the writing of the manuscript. All authors had full access to all study data.

## Results

The initial dataset downloaded on September 27, 2010 included 96,346 clinical trials registered with ClinicalTrials.gov. A total of 40,970 interventional trials were registered from October 1, 2007, after enactment of mandatory registration in September 27, 2007; of these, 3570 (9%) were defined as the ID trials dataset (Figure 1). This was similar to the number of cardiovascular medicine (n = 3437 [8%]) and mental health trials (n = 3695 [9%]) but less than the number of oncology trials (n = 8992 [22%]) [6].

**Figure 3:**
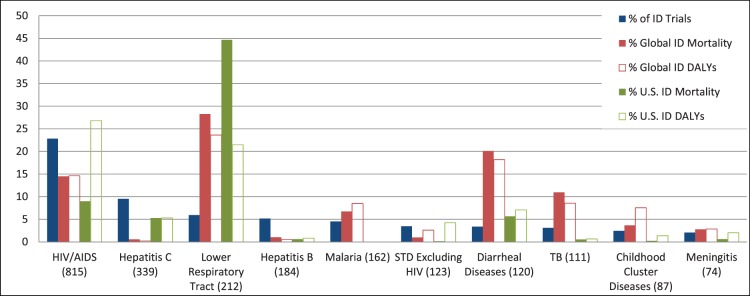
The percentage of selected infectious disease (ID) subcategories in the ID Trials Dataset as compared with the burden of disease in the United States and globally. Number of trials per category is in parentheses. Percent mortality and percentage of disability-adjusted life years (DALYs) are defined relative to total communicable disease-attributable mortality and DALYs lost, respectively. Maternal and perinatal infections were excluded.

Of 3570 ID trials, 3207 (90%) were assigned to 1 subcategory, 329 (9%) were assigned to 2 subcategories, and less than 1% were assigned to more than 2 subcategories. The distribution of trials across 58 ID subcategories is presented in Figure 2. The representation of certain disease subcategories within the ClinicalTrials.gov registry is compared with ID-related mortality and disability (as defined by the WHO Global Burden of Disease) in Figure 3. Based on their frequency in the database and their global impact, 5 subcategories were chosen for more detailed characterization: HIV-AIDS, hepatitis C virus, LRTIs, malaria, and tuberculosis. “Treatment” was the primary purpose in the majority of both ID and non-ID trials (53% and 77%, respectively; Table 1). However, the difference in their relative frequencies was due to the greater number of prevention-oriented trials seen in ID (38% vs. 8%). Further, a greater number of biological interventions was observed among ID trials (29% vs. 5% in non-ID trials), nearly all of which involved vaccine trials. Procedural interventions and devices were under-represented among ID trials (4% vs. 11% for non-ID trials, and 3% vs. 10% for non-ID trials, respectively).

**Table 1:** Characteristics of all non-infectious disease (ID) studies, all ID Studies, and HIV-AIDS, hepatitis C, malaria, and tuberculosis studies registered with ClinicalTrials.gov from October 2007–September 2010.

	Study Focus					
Parameter	Non-ID	All ID	HIV-AIDS	Hepatitis C	Malaria	Tuberculosis
Primary purpose, N	34803	3396	770	309	152	103
Treatment	26820 (77.1%)	1785 (52.6%)	478 (62.1%)	276 (89.3%)	70 (46.1%)	56 (54.4%)
Prevention	2847 (8.2%)	1305 (38.4%)	201 (26.1%)	11 (3.6%)	60 (39.5%)	27 (26.2%)
Other purpose	5136 (14.8%)	306 (9.0%)	91 (11.8%)	22 (7.1%)	22 (14.5%)	20 (19.4%)
Type of intervention, N	37400	3570	815	339	162	111
Drug	22825 (61.0%)	1926 (53.9%)	503 (61.7%)	295 (87.0%)	107 (66.0%)	64 (57.7%)
Procedure	3962 (10.6%)	142 (4.0%)	19 (2.3%)	8 (2.4%)	5 (3.1%)	5 (4.5%)
Biological/vaccine	1925 (5.1%)	1023 (28.7%)	103 (12.6%)	37 (10.9%)	27 (16.7%)	33 (29.7%)
Behavioral	3091 (8.3%)	216 (6.1%)	151 (18.5%)	13 (3.8%)	2 (1.2%)	5 (4.5%)
Device	3701 (9.9%)	98 (2.7%)	13 (1.6%)	2 (0.6%)	7 (4.3%)	1 (0.9%)
Other intervention	6899 (18.4%)	467 (13.1%)	92 (11.3%)	27 (8.0%)	32 (19.8%)	9 (8.1%)
Vaccine		966 (27.1%)	92 (11.3%)	6 (1.8%)	24 (14.8%)	28 (25.2%)
Lead sponsor, N	37400	3570	815	339	162	111
Industry	13774 (36.8%)	1550 (43.4%)	179 (22.0%)	212 (62.5%)	25 (15.4%)	20 (18.0%)
NIH	895 (2.4%)	211 (5.9%)	111 (13.6%)	12 (3.5%)	8 (4.9%)	6 (5.4%)
U.S. federal	481 (1.3%)	70 (2.0%)	8 (1.0%)	6 (1.8%)	15 (9.3%)	4 (3.6%)
Govt.-non-U.S.	145 (0.4%)	100 (2.8%)	44 (5.4%)	6 (1.8%)	4 (2.5%)	14 (12.6%)
Acad./hosp.	13185 (35.3%)	1309 (36.7%)	339 (41.6%)	84 (24.8%)	73 (45.1%)	53 (47.7%)
Consortium	106 (0.3%)	113 (3.2%)	79 (9.7%)	4 (1.2%)	13 (8.0%)	6 (5.4%)
Other	8814 (23.6%)	217 (6.1%)	55 (6.7%)	15 (4.4%)	24 (14.8%)	8 (7.2%)
Funding source, N	37400	3570	815	339	162	111
Industry	17013 (45.5%)	1824 (51.1%)	293 (36.0%)	230 (67.8%)	31 (19.1%)	30 (27.0%)
NIH	3185 (8.5%)	353 (9.9%)	191 (23.4%)	17 (5.0%)	17 (10.5%)	12 (10.8%)
Other	17202 (46.0%)	1393 (39.0%)	331 (40.6%)	92 (27.1%)	114 (70.4%)	69 (62.2%)
Trial facility, N	34283	3237	741	274	156	105
Single facility	22887 (66.8%)	1901 (58.7%)	466 (62.9%)	127 (46.4%)	122 (78.2%)	80 (76.2%)
Multiple facilities	11396 (33.2%)	1336 (41.3%)	275 (37.1%)	147 (53.6%)	34 (21.8%)	25 (23.8%)
Enrollment, N	36839	3527	804	335	156	111
Median (IQR)	60 (30, 160)	125 (45, 400)	66 (30, 240)	60 (30, 150)	241 (48, 1075)	82 (36, 300)
Sex/age, N	37400	3570	815	339	162	111
Female	3502 (9.4%)	228 (6.4%)	73 (9.0%)	4 (1.2%)	20 (12.3%)	1 (0.9%)
Male	2119 (5.7%)	109 (3.1%)	50 (6.1%)	8 (2.4%)	4 (2.5%)	5 (4.5%)
Both	31779 (85.0%)	3233 (90.6%)	692 (84.9%)	327 (96.5%)	138 (85.2%)	105 (94.6%)
Children only^a^	2281 (6.1%)	601 (16.8%)	46 (5.6%)	0 (0.0%)	52 (32.1%)	8 (7.2%)
Excludes elderly^b^	11330 (30.3%)	1717 (48.1%)	339 (41.6%)	161 (47.5%)	118 (72.8%)	62 (55.9%)
Masking/blinding	36332	3539	808	334	160	111
Open	20266 (55.8%)	1968 (55.6%)	548 (67.8%)	206 (61.7%)	122 (76.3%)	74 (66.7%)
Single-blind	4140 (11.4%)	317 (9.0%)	63 (7.8%)	8 (2.4%)	7 (4.4%)	4 (3.6%)
Double-blind	11926 (32.8%)	1254 (35.4%)	197 (24.4%)	120 (35.9%)	31 (19.4%)	33 (29.7%)
Allocation, N	35742	3498	795	328	153	109
Randomized	24458 (68.4%)	2569 (73.4%)	568 (71.4%)	223 (68.0%)	111 (72.5%)	73 (67.0%)
Nonrandomized	11284 (31.6%)	929 (26.6%)	227 (28.6%)	105 (32.0%)	42 (27.5%)	36 (33.0%)
Number of arms, N	35786	3457	786	312	157	102
1	11812 (33.0%)	764 (22.1%)	192 (24.4%)	85 (27.2%)	24 (15.3%)	19 (18.6%)
2	17467 (48.8%)	1738 (50.3%)	418 (53.2%)	107 (34.3%)	81 (51.6%)	47 (46.1%)
3	3627 (10.1%)	474 (13.7%)	94 (12.0%)	48 (15.4%)	26 (16.6%)	15 (14.7%)
4	1830 (5.1%)	257 (7.4%)	45 (5.7%)	32 (10.3%)	16 (10.2%)	7 (6.9%)
5 or more	1050 (2.9%)	224 (6.5%)	37 (4.7%)	40 (12.8%)	10 (6.4%)	14 (13.7%)
Comparator, N	31834	3261	733	292	149	98
Active	13367 (42.0%)	1447 (44.4%)	334 (45.6%)	101 (34.6%)	79 (53.0%)	32 (32.7%)
Placebo	8658 (27.2%)	773 (23.7%)	138 (18.8%)	67 (22.9%)	16 (10.7%)	25 (25.5%)
Phase, N	37400	3570	815	339	162	111
Phase 0	300 (0.8%)	16 (0.4%)	6 (0.7%)	1 (0.3%)	3 (1.9%)	0 (0.0%)
Phase 1	5691 (15.2%)	531 (14.9%)	141 (17.3%)	93 (27.4%)	28 (17.3%)	30 (27.0%)
Phase 1/Phase 2	1960 (5.2%)	145 (4.1%)	37 (4.5%)	20 (5.9%)	11 (6.8%)	3 (2.7%)
Phase 2	7724 (20.7%)	760 (21.3%)	164 (20.1%)	96 (28.3%)	12 (7.4%)	29 (26.1%)
Phase 2/Phase 3	957 (2.6%)	98 (2.7%)	21 (2.6%)	5 (1.5%)	9 (5.6%)	0 (0.0%)
Phase 3	5489 (14.7%)	708 (19.8%)	105 (12.9%)	29 (8.6%)	28 (17.3%)	12 (10.8%)
Phase 4	4906 (13.1%)	653 (18.3%)	169 (20.7%)	61 (18.0%)	37 (22.8%)	16 (14.4%)
N/A	10373 (27.7%)	659 (18.5%)	172 (21.1%)	34 (10.0%)	34 (21.0%)	21 (18.9%)
Overall status, N	37400	3570	815	339	162	111
Not yet recruiting	3337 (8.9%)	388 (10.9%)	83 (10.2%)	23 (6.8%)	34 (21.0%)	9 (8.1%)
Recruiting	16959 (45.3%)	1295 (36.3%)	353 (43.3%)	134 (39.5%)	53 (32.7%)	58 (52.3%)
Active, not recruiting	4430 (11.8%)	555 (15.5%)	163 (20.0%)	62 (18.3%)	17 (10.5%)	11 (9.9%)
Completed	11041 (29.5%)	1224 (34.3%)	196 (24.0%)	113 (33.3%)	54 (33.3%)	30 (27.0%)
Terminated	1633 (4.4%)	108 (3.0%)	20 (2.5%)	7 (2.1%)	4 (2.5%)	3 (2.7%)
DMC, N	37400	3570	815	339	162	111
Has DMC	12388 (33.1%)	1256 (35.2%)	306 (37.5%)	115 (33.9%)	86 (53.1%)	59 (53.2%)
No DMC	18454 (49.3%)	1517 (42.5%)	385 (47.2%)	135 (39.8%)	56 (34.6%)	32 (28.8%)
DMC missing	6558 (17.5%)	797 (22.3%)	124 (15.2%)	89 (26.3%)	20 (12.3%)	20 (18.0%)
Regional distribution^c^, N	34283	3237	741	274	156	105
Africa	520 (1.5%)	297 (9.2%)	119 (16.1%)	4 (1.5%)	88 (56.4%)	37 (35.2%)
Central America	344 (1.0%)	112 (3.5%)	33 (4.5%)	33 (12.0%)	0 (0.0%)	0 (0.0%)
East Asia	3239 (9.4%)	296 (9.1%)	18 (2.4%)	40 (14.6%)	1 (0.6%)	11 (10.5%)
Europe	10371 (30.3%)	940 (29.0%)	178 (24.0%)	95 (34.7%)	15 (9.6%)	28 (26.7%)
Middle East	1452 (4.2%)	93 (2.9%)	3 (0.4%)	18 (6.6%)	0 (0.0%)	3 (2.9%)
North America	20103 (58.6%)	1478 (45.7%)	409 (55.2%)	148 (54.0%)	20 (12.8%)	18 (17.1%)
North Asia	780 (2.3%)	49 (1.5%)	11 (1.5%)	1 (0.4%)	0 (0.0%)	5 (4.8%)
Pacifica	979 (2.9%)	108 (3.3%)	15 (2.0%)	23 (8.4%)	3 (1.9%)	1 (1.0%)
South America	1326 (3.9%)	189 (5.8%)	41 (5.5%)	18 (6.6%)	8 (5.1%)	8 (7.6%)
South Asia	752 (2.2%)	124 (3.8%)	12 (1.6%)	3 (1.1%)	7 (4.5%)	8 (7.6%)
Southeast Asia	702 (2.0%)	144 (4.4%)	43 (5.8%)	2 (0.7%)	17 (10.9%)	9 (8.6%)
Unknown	3117 (8.3%)	333 (9.3%)	74 (9.1%)	65 (19.2%)	6 (3.7%)	6 (5.4%)

The denominator for each variable is the number of trials reporting such data. “Other Purpose” includes “Diagnostic”, “Supportive Care”, “Screening”, “Health Services Research”, and “Basic Science”. For Intervention type, the numerator is the number of trials with at least 1 intervention of this type. A study with multiple interventions may be represented in more than 1 intervention type; hence, cumulative percentage will exceed 100%. “Other Intervention” includes “Radiation”, “Dietary Supplement”, and “Genetic”. The “Comparator” variable identifies the number of trials designating that particular comparator. Since a study may have both a placebo and an active comparator arm, the cumulative percentage may exceed 100%. The “Recruiting” variable under “Overall Status” includes trials recruiting by invitation. “Terminated” includes trials that have been terminated, suspended, or withdrawn. The numerator in the regional distribution variable is the number of trials with at least 1 study site in that respective region. A multisite study may be represented in more than 1 region; hence the cumulative percentage will exceed 100%. Abbreviations: DMC, data monitoring committee; ID, infectious diseases; IQR, interquartile range; NIH = US National Institutes of Health.

a Children defined as ≤18 years of age.

b Elderly defined as >65 years of age.

c Individual countries by region are available at: http://www.clinicaltrials.gov/ct2/search/browse?brwse=locn_cat.

Among 3513 ID clinical trials providing enrollment information, 34% reported actual enrollment and 66% reported anticipated enrollment. Infectious disease trials tended to be larger, with a median (anticipated or actual) enrollment of 125 subjects (interquartile range [IQR], 45–400) vs. 60 (IQR, 30–160) for non-ID studies. Only 3% of non-ID trials enrolled (anticipated or actual) more than 1000 subjects, in contrast with 10% of ID trials. The largest trials–those enrolling (anticipated or actual) >10,000 subjects–were more frequently represented within the ID trials dataset and accounted for 31% (35/109) of all such studies regardless of specialty. HIV-AIDS (n = 7), malaria (n = 5) and influenza vaccine (n = 5) trials accounted for half of all ID trials enrolling >10,000 subjects. Prevention-oriented trials were larger than treatment-oriented trials (median 240; IQR, 98–680 vs. median 90; IQR, 36–236) (Table S1). However, even after excluding the larger prevention studies, ID trials were still notably larger than all non-ID studies (median 90; IQR, 36–236 vs. median 60; IQR 30–160).

ID trials were more likely than non-ID trials to be restricted to children (≤18 years; 17% vs. 6%) yet also more likely to exclude elderly persons (>65 years; 48% vs. 30%). Among ID trials, those with a primary purpose of prevention were more likely to restrict enrollment to children than those with a treatment or other purpose (27% vs. 11%; χ^2^ p-value<0.0001) (Table S1). Similarly, ID vaccine trials were more likely to restrict enrollment to children than non-vaccine trials (30% vs. 12%; χ^2^ p-value <0.0001). However, even among trials where the primary purpose was not prevention, trials that restricted enrollment to children were more common among ID trials than non-ID trials (10.9% vs. 5.6%; χ^2^ p-value <0.0001). The majority of ID trials were randomized (73% vs. 68%), although both ID and non-ID trials had the same proportion (56%) of open (i.e., non-blinded) trials. Reporting the presence or absence of a data monitoring committee (DMC) was not required for study registration. Consequently, 22% of studies in the ID trials dataset lacked this information. Among the 2763 studies that reported DMC status, the majority (55%) did not have one–rates similar to those seen in non-ID trials (60%). Among studies reporting DMC status, non-U.S.-based studies were more likely to use DMCs than U.S.-based studies (48% vs. 39%, respectively). However, a large number of trials did not provide DMC data in their registration information (21% and 17% for non-U.S.- and U.S.-based studies, respectively).

**Figure 4:**
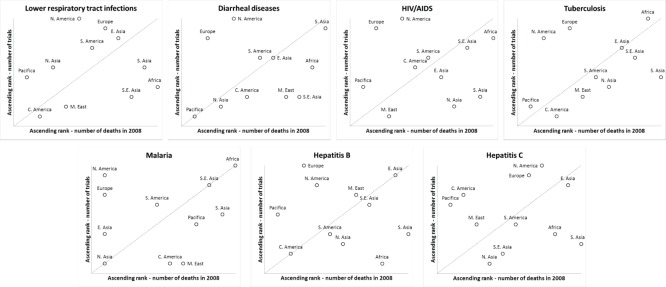
Rank of disease-specific trial frequency vs. disease-related mortality across eleven world regions. Data is presented for 7 categories: LRTI, diarrheal diseases, HIV/AIDS, tuberculosis, malaria, hepatitis B, and hepatitis C. Panels are arrayed in descending order of global disease mortality from left to right and top to bottom. Trial rank was calculated by first identifying all trials for the specified disease and then ranking world regions based on the number of trials including that region. Death rank was calculated using the total number of deaths per region due to the specified disease. The diagonal line is provided for reference. Regions above the line rank higher in terms of number of trials compared to disease-specific mortality. Regions below the line rank lower in terms of number of trials compared to disease-specific mortality. Regions on or close to the line have similar trial and mortality ranks.

North America was the most frequently identified study location for ID studies (46%, Table 1 and Table S2). Compared with non-ID studies, other world regions were more frequently identified in the ID trials dataset; further, these regions were associated with local disease prevalence. For example, studies focusing on malaria and tuberculosis were concentrated in Africa (56% and 35%, respectively) but infrequently located in North America (13% and 17%, respectively). In contrast, 42% of LRTI trials were located in North America and 36% in Europe, but only 6% were located in Africa. We also plotted the geographic distribution of trials relative to the burden of disease across eleven world regions based on rank (Figure 4). For the 7 disease conditions analyzed (LRTI, Diarrheal diseases, HIV/AIDS, Tuberculosis, Malaria, Hepatitis B, and Hepatitis C), North America and Europe were included as study sites disproportionately more frequently than the burden of disease affecting these areas, whereas South Asia was under-represented. No clear patterns emerged for other world regions across the 7 diseases. Some world regions were well matched between the relative number of trials and the burden of that disease, whether high or low (e.g., Southeast Asia and Africa for Malaria, or Central America and East Asia for Hepatitis B).

Trial characteristics, stratified by funding source, are shown in Table S3. Industry-funded studies were more likely to focus on treatment (54%) and have drug-based interventions (57%) compared with NIH-funded studies (38% and 42%, respectively). In contrast, a greater proportion of NIH-funded studies focused on behavioral interventions than did industry-funded studies (26% vs. <1%, respectively). Trials of phase 3 or later accounted for 24% of NIH-funded interventional studies, compared with 44% of industry-funded trials. Trial status also differed by source of funding: more industry-funded trials were identified as completed (45%, vs. 18% for NIH-funded), while more NIH-funded trials were identified as recruiting (45%, vs. 26% for industry-funded). The percentage of trials with a DMC also differed by funding source; 40% of industry-funded trials included a DMC, compared with 61% of NIH-funded trials. Study size and other characteristics were otherwise comparable between industry- and NIH-funded trials.

Several trial characteristics differed based on U.S vs. non-U.S. trial locations (Table S4). Non-U.S.-based trials focused on prevention strategies more often than U.S.-based ones (43% vs. 36%). The NIH was the lead sponsor for 15% of U.S.-based trials, but only 1% of non-U.S.-based trials. Otherwise, there was a similar distribution of sponsorship among industry and academic/hospital-based institutions. Trials tended to be larger in non-U.S.-based studies, with a median enrollment of 150 persons (IQR, 52–408) vs. 75 persons (IQR, 32–260) for U.S.-based trials. Non-U.S.-based trials were more likely to restrict enrollment to children (21%, vs. 10% of U.S.-based trials). Fewer U.S.-based trials were identified as phase 3 or later (32%, vs. 55% of non-U.S.-based trials).

## Discussion

An overview of all ClinicalTrials.gov trials was recently published [6], although this study is the first to specifically characterize the subset of ID trials within the ClinicalTrials.gov registry. While there are more comprehensive registries, such as the International Clinical Trials Registry Platform (ICTRP) managed by the WHO, we chose to focus on ClinicalTrials.gov because of the tools available for its characterization, notably the AACT database facilitated by the CTTI. A manual review of several ID-specific disease states revealed that about 80% of trials in the ICTRP were also registered in ClinicalTrials.gov (data not shown). Therefore, although our study does not characterize all ID trials, it provides a representative contemporary sample from which to describe the overall state of ID clinical trials.

We found that ID trials were heterogeneous, but also shared some interesting specialty-specific themes. The emphasis of ID clinical trials on prevention strategies, primarily vaccine interventions, highlights the public health direction of the field and suggests that further investment in ID trials may facilitate population-level impact. We did note that prevention trials more frequently included sites outside of North America. We could not identify a clear reason for this although one factor considered to play prominently in this finding is the heavy emphasis on vaccines in prevention trials. Vaccination programs as part of routine clinical care in the U.S. are robust compared to other countries and regions. As such, non-U.S. based sites investigating vaccines represent opportunities both for mutualistic benefit–to the trial and to the population. Furthermore, this focus on prevention through vaccination was hypothesized to be one reason why ID trials tend to restrict enrollment to pediatric subjects more frequently than non-ID trials. We found that among ID trials, vaccine and prevention trials were significantly more likely to restrict enrollment to children than non-vaccine or non-prevention trials. However, among all trials where prevention was not the primary purpose, ID trials were still more likely to restrict enrollment to children than non-ID trials. Prevention-focused trials may still contribute to the higher rates of pediatric enrollment, although there are likely to be other explanatory factors such as the greater burden of infectious diseases carried by children as compared with other chronic or non-communicable diseases. Conversely, ID trials tended to exclude elderly subjects more often than non-ID trials. This may again relate to the disproportionate burden of communicable diseases affecting children. However, this exclusion of the elderly was seen in ID subcategories that do affect elderly persons with a disproportionately high burden of morbidity and mortality. Careful consideration should be given before excluding elderly subjects in future study designs. We note that this analysis of the ClinicalTrials.gov registry likely underestimates the prevalence of preventative strategies, because the legal requirement for registration with ClinicalTrials.gov exempts phase 1 trials, trials not involving a drug, biologic, or device, and trials not under U.S. jurisdiction. This applies not only to ID trials but likely extends to other trial categories as well.

We also found that ID trials tended to be larger than trials in other specialties, both in terms of median actual or anticipated subject enrollment, and in representation of studies enrolling (actual or anticipated) >10,000 subjects. Despite the larger median size of ID studies, we observed considerable heterogeneity in this regard. For example, the median size of 4 trachoma trials was 8438 subjects; 40 trials focusing on *Haemophilus* had a median enrollment of 600 subjects; and 111 trials focusing on STDs (excluding HIV) had a median enrollment of 400 subjects. This contrasts with several other subcategories with a concentration of small trials: the median size for HIV-AIDS trials was 66 subjects; for HCV trials, 60 subjects. Clinical trialists continue to face ongoing challenges with regard to the ability to enroll adequate numbers of research participants [16], but this issue is not universally applicable across the field of ID. We cannot, however, determine whether differences in study size reflect differences in prioritization, funding, or other resources. Though these factors may be playing a role, it is also true that some diseases and trials require fewer participants to meet the specified objectives – scientific, epidemiological, statistical, or otherwise. This consideration applies to different infectious diseases as well as to non-infectious conditions. The results and methodology presented here suggest an opportunity to consolidate clinical research efforts for maximal impact.

This snapshot of ID clinical trials may also be useful for guiding future funding and policy decision-making. In our review, we found that frequency of particular ID trial subcategories did not correlate directly with their global or U.S. health impact. For example, although HIV-AIDS trials represented 23% of the ID trials dataset, it accounts for 15% of global communicable disease-related mortality and 9% of U.S. communicable disease-related mortality (Figure 3). Trials focusing on hepatitis C also constituted a larger percentage of the ID trials dataset than the estimated global mortality. In contrast, LRTI studies made up only 6% of ID studies, but LRTI ranks as the most common cause of global and U.S. communicable disease-related deaths (28% and 45%, respectively) [14], [15]. Diarrheal diseases also contribute a significant burden of global communicable disease-related mortality and disability [13]–[15] but were not substantially represented in the ClinicalTrials.gov ID portfolio. This discrepancy is cited as one reason for the failure to achieve Millennium Development Goals to reduce childhood death due to diarrheal disease [17]. The greatest discrepancy between representation in the ID trials dataset and communicable disease-related mortality rates was observed with the category of “High-Intensity Intestinal Nematode Infections”. This most prevalent of ID-related conditions affects more than 150 million people worldwide annually [13]; however, only 12 such trials (<1%) were identified in the ID trials dataset. Another common clinical problem, particularly in economically developed nations, is prosthetic joint infection (PJI) and osteomyelitis [18]–[20]. Although optimal treatment of PJI and osteomyelitis is a common challenge for many ID practitioners, we identified only 10 trials focusing on these infections.

The lack of correlation between diseases with high global mortality and the number of clinical trials targeting these infections was striking, and suggests a need for further emphasis on research in high-mortality clinical syndromes. Even among infections that cause high morbidity in the United States, such as PJI and osteomyelitis, we found few trials focused on these conditions in the ClinicalTrials.gov registry. The absence of promising therapies may explain why trials for a particular disease are under-represented, in which case future studies should emphasize basic science and drug discovery. For example, the relatively high prevalence of hepatitis C trials may be related to the recent discovery of new protease inhibitor therapies.

Despite the significant value provided by the ID trials dataset in this analysis, this resource has limitations. First, ClinicalTrials.gov was primarily designed as a public repository for research trials and was not intended to support aggregate analysis. Second, because the methodology developed to annotate the ClinicalTrials.gov database by clinical specialty relied on a group of experts drawn exclusively from a single institution (Duke University), further validation would be appropriate. Third, this analysis includes interventional trials for which the FDA requires registration, which may introduce a bias toward trials involving therapeutics rather than other intervention strategies. However, publishers who have adopted the International Committee of Medical Journal Editors’ Uniform Requirements mandate registration of all interventional studies, regardless of intervention type, phase, or location [4]. Despite the FDAAA requirement to register all applicable interventional studies within 21 days of a study’s start, many fail to do so in a timely manner [21]. As a result, there are likely to be some trials begun within our October 2007–September 2010 window that were not registered and therefore are not included here.

Our analysis also excludes observational and other non-interventional epidemiologic studies and thus we present a cross-section of ID clinical trials, not a comprehensive review of all ID-related clinical research. When study sponsors or investigators register a clinical trial in ClinicalTrials.gov, not all fields are mandatory. For example, registrants were not required to specify whether a DMC was involved in the trial. This limits our interpretation of the available data, given how frequently this particular parameter was missing. It also suggests that ClinicalTrials.gov may need to revise the list of mandatory fields so as to provide a more robust assessment of a given clinical trial.

Another caveat to the interpretation of our findings is that the number of trials or participants within a given disease area is not a surrogate measure for resource investment. Some small trials may indeed be very expensive and some large trials may be relatively inexpensive. Unfortunately, ClinicalTrials.gov does not record data on financial or resource expenditures. Furthermore, research into some disease states may not require ClinicalTrials.gov registration and would therefore appear to be underrepresented. This includes health systems research such as implementation research and quality improvement research.

Our analysis of ClinicalTrials.gov revealed that ID trials are well-represented as a proportion of the overall clinical trials enterprise, tend to be larger than non-ID trials, and have a greater representation outside of the United States than do other specialties. However, there is considerable variation across ID trials, much of which reflects the heterogeneity of infectious diseases themselves. Our analysis highlights discrepancies between the number and quality of trials in some disease states relative to the global burden of those diseases, and may prompt examination of how best to prioritize and coordinate research funding both within and across national boundaries. Data generated from high-quality clinical trials not only improves disease management but also informs clinical practice guidelines. The results presented here should inform improvements in clinical research methodology, focus resources more deliberately, and serve as a launchpad for establishing future clinical research priorities.

## Supporting Information

Table S1 **Characteristics of ID studies stratified by primary purpose, October 2007–September 2010.**
(DOC)

Table S2 **Frequency and percentage of ID trials based on region and country.**
(DOCX)

Table S3 **Infectious disease clinical trial attributes by funding source: Industry, NIH, or Other.**
(DOCX)

Table S4 **Infectious disease clinical trial attributes by study location: U.S., Non-U.S., and Both.**
(DOCX)

Appendix S1 **List of ID Terms Used to Identify Potential Infectious Disease Trials from the ClinicalTrials.gov Registry.**
(DOC)

Diagram S1 **PRISMA diagram.**
(DOC)

Checklist S1 **PRISMA checklist.**
(DOC)
