# Supplementary material for: The State of Infectious Diseases Clinical Trials: A Systematic Review of ClinicalTrials.gov
Source: PLoS One. 2013 Oct 16;8(10):e77086. doi: 10.1371/journal.pone.0077086 (PMC3797691; doi:10.1371/journal.pone.0077086)
Supplement: Table S1 — Characteristics of ID studies stratified by primary purpose, October 2007–September 2010. (DOC) [file pone.0077086.s001.doc]

| **Table S1. Characteristics of ID studies stratified by primary purpose, October 2007 - September 2010.** | | | | |
| --- | --- | --- | --- | --- |
|  | **Primary purpose** | | | |
| **Parameter** | **All ID**  **(N=3570)** | **ID Prevention**  **(N=1305)** | **ID Treatment**  **(N=1785)** | **ID Other**  **(N=306)** |
| Primary purpose, N | 3396 | 1305 | 1785 | 306 |
| Treatment | 1785 (52.6%) | 0 (0.0%) | 1785 (100.0%) | 0 (0.0%) |
| Prevention | 1305 (38.4%) | 1305 (100.0%) | 0 (0.0%) | 0 (0.0%) |
| Other purpose | 306 (9.0%) | 0 (0.0%) | 0 (0.0%) | 306 (100.0%) |
| Type of intervention, N | 3570 | 1305 | 1785 | 306 |
| Drug | 1926 (53.9%) | 237 (18.2%) | 1491 (83.5%) | 90 (29.4%) |
| Procedure | 142 (4.0%) | 28 (2.1%) | 68 (3.8%) | 36 (11.8%) |
| Biological/vaccine | 1023 (28.7%) | 834 (63.9%) | 119 (6.7%) | 47 (15.4%) |
| Behavioral | 216 (6.1%) | 106 (8.1%) | 54 (3.0%) | 50 (16.3%) |
| Device | 98 (2.7%) | 34 (2.6%) | 39 (2.2%) | 20 (6.5%) |
| Other intervention | 467 (13.1%) | 162 (12.4%) | 174 (9.7%) | 96 (31.4%) |
| Vaccine | 966 (27.1%) | 847 (64.9%) | 63 (3.5%) | 36 (11.8%) |
| Lead sponsor, N | 3570 | 1305 | 1785 | 306 |
| Industry | 1550 (43.4%) | 644 (49.3%) | 774 (43.4%) | 49 (16.0%) |
| NIH | 211 (5.9%) | 123 (9.4%) | 67 (3.8%) | 15 (4.9%) |
| U.S. federal | 70 (2.0%) | 28 (2.1%) | 37 (2.1%) | 5 (1.6%) |
| Govt.-non-U.S. | 100 (2.8%) | 43 (3.3%) | 45 (2.5%) | 8 (2.6%) |
| Acad./hosp. | 1309 (36.7%) | 358 (27.4%) | 686 (38.4%) | 204 (66.7%) |
| Consortium | 113 (3.2%) | 39 (3.0%) | 61 (3.4%) | 5 (1.6%) |
| Other | 217 (6.1%) | 70 (5.4%) | 115 (6.4%) | 20 (6.5%) |
| Funding source, N | 3570 | 1305 | 1785 | 306 |
| Industry | 1824 (51.1%) | 716 (54.9%) | 939 (52.6%) | 73 (23.9%) |
| NIH | 353 (9.9%) | 164 (12.6%) | 128 (7.2%) | 48 (15.7%) |
| Other | 1393 (39.0%) | 425 (32.6%) | 718 (40.2%) | 185 (60.5%) |
| Trial facility, N | 3237 | 1196 | 1600 | 281 |
| Single facility | 1901 (58.7%) | 659 (55.1%) | 905 (56.6%) | 217 (77.2%) |
| Multiple facilities | 1336 (41.3%) | 537 (44.9%) | 695 (43.4%) | 64 (22.8%) |
| Enrollment |  |  |  |  |
| N | 3527 | 1297 | 1776 | 296 |
| Median (IQR) | 125 (45, 400) | 240 (98, 680) | 90 (36, 236) | 140 (39, 500) |
| Sex/age, N | 3570 | 1305 | 1785 | 306 |
| Female | 228 (6.4%) | 122 (9.3%) | 77 (4.3%) | 21 (6.9%) |
| Male | 109 (3.1%) | 51 (3.9%) | 32 (1.8%) | 18 (5.9%) |
| Both | 3233 (90.6%) | 1132 (86.7%) | 1676 (93.9%) | 267 (87.3%) |
| Restricted to children^a^ | 601 (16.8%) | 351 (26.9%) | 199 (11.1%) | 29 (9.5%) |
| Excludes elderly^b^ | 1717 (48.1%) | 804 (61.6%) | 680 (38.1%) | 130 (42.5%) |
| Masking/blinding | 3539 | 1301 | 1782 | 295 |
| Open | 1968 (55.6%) | 624 (48.0%) | 1023 (57.4%) | 207 (70.2%) |
| Single-blind | 317 (9.0%) | 157 (12.1%) | 122 (6.8%) | 31 (10.5%) |
| Double-blind | 1254 (35.4%) | 520 (40.0%) | 637 (35.7%) | 57 (19.3%) |
| Allocation, N | 3498 | 1294 | 1766 | 284 |
| Randomized | 2569 (73.4%) | 987 (76.3%) | 1310 (74.2%) | 178 (62.7%) |
| Nonrandomized | 929 (26.6%) | 307 (23.7%) | 456 (25.8%) | 106 (37.3%) |
| Number of arms, N | 3457 | 1286 | 1725 | 292 |
| 1 | 764 (22.1%) | 222 (17.3%) | 403 (23.4%) | 87 (29.8%) |
| 2 | 1738 (50.3%) | 612 (47.6%) | 912 (52.9%) | 151 (51.7%) |
| 3 | 474 (13.7%) | 204 (15.9%) | 226 (13.1%) | 26 (8.9%) |
| 4 | 257 (7.4%) | 127 (9.9%) | 111 (6.4%) | 15 (5.1%) |
| 5 or more | 224 (6.5%) | 121 (9.4%) | 73 (4.2%) | 13 (4.5%) |
| Comparator, N | 3261 | 1226 | 1620 | 266 |
| Active comparator arm | 1447 (44.4%) | 496 (40.5%) | 797 (49.2%) | 102 (38.3%) |
| Placebo comparator arm | 773 (23.7%) | 308 (25.1%) | 399 (24.6%) | 45 (16.9%) |
| Phase, N | 3570 | 1305 | 1785 | 306 |
| Phase 0 | 16 (0.4%) | 3 (0.2%) | 6 (0.3%) | 5 (1.6%) |
| Phase 1 | 531 (14.9%) | 196 (15.0%) | 224 (12.5%) | 51 (16.7%) |
| Phase 1/Phase 2 | 145 (4.1%) | 60 (4.6%) | 71 (4.0%) | 4 (1.3%) |
| Phase 2 | 760 (21.3%) | 304 (23.3%) | 423 (23.7%) | 13 (4.2%) |
| Phase 2/Phase 3 | 98 (2.7%) | 40 (3.1%) | 53 (3.0%) | 5 (1.6%) |
| Phase 3 | 708 (19.8%) | 334 (25.6%) | 342 (19.2%) | 19 (6.2%) |
| Phase 4 | 653 (18.3%) | 177 (13.6%) | 414 (23.2%) | 42 (13.7%) |
| N/A | 659 (18.5%) | 191 (14.6%) | 252 (14.1%) | 167 (54.6%) |
| Overall status, N | 3570 | 1305 | 1785 | 306 |
| Not yet recruiting | 388 (10.9%) | 147 (11.3%) | 192 (10.8%) | 27 (8.8%) |
| Recruiting | 1295 (36.3%) | 341 (26.1%) | 745 (41.7%) | 144 (47.1%) |
| Active, not recruiting | 555 (15.5%) | 258 (19.8%) | 242 (13.6%) | 39 (12.7%) |
| Completed | 1224 (34.3%) | 529 (40.5%) | 535 (30.0%) | 90 (29.4%) |
| Terminated | 108 (3.0%) | 30 (2.3%) | 71 (4.0%) | 6 (2.0%) |
| DMC, N | 3570 | 1305 | 1785 | 306 |
| Has DMC | 1256 (35.2%) | 438 (33.6%) | 685 (38.4%) | 95 (31.0%) |
| No DMC | 1517 (42.5%) | 441 (33.8%) | 799 (44.8%) | 177 (57.8%) |
| DMC missing | 797 (22.3%) | 426 (32.6%) | 301 (16.9%) | 34 (11.1%) |
| Regional distribution^c^, N | 3237 | 1196 | 1600 | 281 |
| Africa | 297 (9.2%) | 106 (8.9%) | 145 (9.1%) | 36 (12.8%) |
| Central America | 112 (3.5%) | 26 (2.2%) | 82 (5.1%) | 2 (0.7%) |
| East Asia | 296 (9.1%) | 105 (8.8%) | 170 (10.6%) | 12 (4.3%) |
| Europe | 940 (29.0%) | 373 (31.2%) | 451 (28.2%) | 72 (25.6%) |
| Middle East | 93 (2.9%) | 22 (1.8%) | 62 (3.9%) | 5 (1.8%) |
| North America | 1478 (45.7%) | 488 (40.8%) | 778 (48.6%) | 129 (45.9%) |
| North Asia | 49 (1.5%) | 7 (0.6%) | 38 (2.4%) | 2 (0.7%) |
| Pacifica | 108 (3.3%) | 37 (3.1%) | 64 (4.0%) | 2 (0.7%) |
| South America | 189 (5.8%) | 50 (4.2%) | 118 (7.4%) | 12 (4.3%) |
| South Asia | 124 (3.8%) | 43 (3.6%) | 73 (4.6%) | 6 (2.1%) |
| Southeast Asia | 144 (4.4%) | 51 (4.3%) | 72 (4.5%) | 9 (3.2%) |
| Unknown | 333 (9.3%) | 109 (8.4%) | 185 (10.4%) | 25 (8.2%) |
| The denominator for each variable is the number of trials reporting such data. “Other Purpose” includes “Diagnostic,” “Supportive Care,” “Screening,” “Health Services Research,” and “Basic Science.” For Intervention type, the numerator is the number of trials with at least 1 intervention of this type. A study with multiple interventions may be represented in more than 1 intervention type; hence, cumulative percentage will exceed 100%. “Other Intervention” includes “Radiation,” “Dietary Supplement,” and “Genetic.” The “Comparator” variable identifies the number of trials designating that particular comparator. Since a study may have both a placebo and an active comparator arm, the cumulative percentage may exceed 100%. The “Recruiting” variable under “Overall Status” includes trials recruiting by invitation. “Terminated” includes trials that have been terminated, suspended, or withdrawn. The numerator in the regional distribution variable is the number of trials with at least 1 study site in that respective region. A multisite study may be represented in more than 1 region; hence the cumulative percentage will exceed 100%. Abbreviations: DMC, data monitoring committee; ID, infectious diseases; IQR, interquartile range; NIH = US National Institutes of Health.  ^a^Children defined as ≤18 years of age. ^b^Elderly defined as >65 years of age. ^c^Individual countries by region are available at: <http://www.clinicaltrials.gov/ct2/search/browse?brwse=locn_cat>. | | | | |
