# Supplementary material for: The State of Infectious Diseases Clinical Trials: A Systematic Review of ClinicalTrials.gov
Source: PLoS One. 2013 Oct 16;8(10):e77086. doi: 10.1371/journal.pone.0077086 (PMC3797691; doi:10.1371/journal.pone.0077086)
Supplement: Table S2 — Frequency and percentage of ID trials based on region and country. (DOCX) [file pone.0077086.s002.docx]

| **Table S2. Frequency and percentage of ID trials based on region and country.** | | |
| --- | --- | --- |
| **Region and country** | **Number of ID studies** | **Percentage of studies**  **(Total N=3237)*** |
| **Africa** |  |  |
| Angola | 1 | 0.03 |
| Benin | 6 | 0.19 |
| Botswana | 8 | 0.25 |
| Burkina Faso | 22 | 0.68 |
| Cameroon | 7 | 0.22 |
| Congo | 6 | 0.19 |
| Cote D'Ivoire | 6 | 0.19 |
| Egypt | 8 | 0.25 |
| Ethiopia | 13 | 0.40 |
| Gabon | 10 | 0.31 |
| Gambia | 6 | 0.19 |
| Ghana | 10 | 0.31 |
| Guinea-Bissau | 3 | 0.09 |
| Kenya | 41 | 1.27 |
| Liberia | 2 | 0.06 |
| Madagascar | 1 | 0.03 |
| Malawi | 12 | 0.37 |
| Mali | 15 | 0.46 |
| Morocco | 1 | 0.03 |
| Mozambique | 3 | 0.09 |
| Niger | 3 | 0.09 |
| Nigeria | 5 | 0.15 |
| Rwanda | 5 | 0.15 |
| Senegal | 7 | 0.22 |
| South Africa | 92 | 2.84 |
| Sudan | 5 | 0.15 |
| Tanzania | 28 | 0.86 |
| Tunisia | 1 | 0.03 |
| Uganda | 45 | 1.39 |
| Zambia | 16 | 0.49 |
| Zimbabwe | 8 | 0.25 |
| **Central America** |  |  |
| Belize | 1 | 0.03 |
| Costa Rica | 12 | 0.37 |
| Cuba | 3 | 0.09 |
| Dominican Republic | 9 | 0.28 |
| Guatemala | 10 | 0.31 |
| Haiti | 1 | 0.03 |
| Honduras | 3 | 0.09 |
| Jamaica | 1 | 0.03 |
| Nicaragua | 1 | 0.03 |
| Panama | 9 | 0.28 |
| Puerto Rico | 82 | 2.53 |
| Saint Kitts and Nevis | 1 | 0.03 |
| **Eastern Asia** |  |  |
| China | 113 | 3.49 |
| Hong Kong | 18 | 0.56 |
| Japan | 45 | 1.39 |
| Korea, Republic of | 60 | 1.85 |
| Taiwan | 85 | 2.63 |
| **Europe** |  |  |
| Austria | 57 | 1.76 |
| Belgium | 124 | 3.83 |
| Bosnia and Herzegovina | 3 | 0.09 |
| Bulgaria | 15 | 0.46 |
| Croatia | 7 | 0.22 |
| Czech Republic | 42 | 1.30 |
| Denmark | 36 | 1.11 |
| Estonia | 18 | 0.56 |
| Finland | 40 | 1.24 |
| Former Serbia and Montenegro | 1 | 0.03 |
| France | 214 | 6.61 |
| Germany | 228 | 7.04 |
| Greece | 22 | 0.68 |
| Hungary | 33 | 1.02 |
| Iceland | 2 | 0.06 |
| Ireland | 12 | 0.37 |
| Italy | 123 | 3.80 |
| Latvia | 11 | 0.34 |
| Lithuania | 16 | 0.49 |
| Luxembourg | 1 | 0.03 |
| Netherlands | 83 | 2.56 |
| Norway | 13 | 0.40 |
| Poland | 67 | 2.07 |
| Portugal | 20 | 0.62 |
| Romania | 36 | 1.11 |
| Serbia | 4 | 0.12 |
| Slovakia | 15 | 0.46 |
| Slovenia | 4 | 0.12 |
| Spain | 155 | 4.79 |
| Sweden | 48 | 1.48 |
| Switzerland | 63 | 1.95 |
| United Kingdom | 163 | 5.04 |
| **Middle East** |  |  |
| Iran, Islamic Republic of | 8 | 0.25 |
| Israel | 65 | 2.01 |
| Jordan | 2 | 0.06 |
| Lebanon | 5 | 0.15 |
| Oman | 1 | 0.03 |
| Qatar | 2 | 0.06 |
| Saudi Arabia | 4 | 0.12 |
| Turkey | 14 | 0.43 |
| **North America** |  |  |
| Canada | 196 | 6.05 |
| Mexico | 64 | 1.98 |
| United States | 1341 | 41.43 |
| **North Asia** |  |  |
| Armenia | 1 | 0.03 |
| Georgia | 3 | 0.09 |
| Russian Federation | 42 | 1.30 |
| Ukraine | 10 | 0.31 |
| **Pacifica** |  |  |
| American Samoa | 1 | 0.03 |
| Australia | 96 | 2.97 |
| New Zealand | 21 | 0.65 |
| Papua New Guinea | 1 | 0.03 |
| Solomon Islands | 1 | 0.03 |
| **South America** |  |  |
| Argentina | 54 | 1.67 |
| Bolivia | 3 | 0.09 |
| Brazil | 102 | 3.15 |
| Chile | 23 | 0.71 |
| Colombia | 27 | 0.83 |
| Ecuador | 1 | 0.03 |
| Paraguay | 1 | 0.03 |
| Peru | 33 | 1.02 |
| Uruguay | 1 | 0.03 |
| Venezuela | 1 | 0.03 |
| **South Asia** |  |  |
| Afghanistan | 6 | 0.19 |
| Bangladesh | 20 | 0.62 |
| India | 81 | 2.50 |
| Nepal | 2 | 0.06 |
| Pakistan | 15 | 0.46 |
| Sri Lanka | 1 | 0.03 |
| **Southeast Asia** |  |  |
| Cambodia | 3 | 0.09 |
| Indonesia | 7 | 0.22 |
| Lao People's Democratic Republic | 1 | 0.03 |
| Malaysia | 7 | 0.22 |
| Myanmar | 1 | 0.03 |
| Philippines | 21 | 0.65 |
| Singapore | 29 | 0.90 |
| Thailand | 84 | 2.59 |
| Vietnam | 12 | 0.37 |
| *Percentage of 3237 Infectious Diseases studies with region and country information for research sites | | |
