# Supplementary material for: The State of Infectious Diseases Clinical Trials: A Systematic Review of ClinicalTrials.gov
Source: PLoS One. 2013 Oct 16;8(10):e77086. doi: 10.1371/journal.pone.0077086 (PMC3797691; doi:10.1371/journal.pone.0077086)
Supplement: Table S3 — Infectious disease clinical trial attributes by funding source: Industry, NIH, or Other. (DOCX) [file pone.0077086.s003.docx]

| **Table S3. Infectious disease clinical trial attributes by funding source: Industry, NIH, or Other.** | | | |
| --- | --- | --- | --- |
|  | **Funding Source** | | |
| **Parameter** | **Industry funding** | **NIH funding** | **Other funding** |
| Primary purpose, N | 1728 | 340 | 1328 |
| Treatment | 939 (54.3%) | 128 (37.6%) | 718 (54.1%) |
| Prevention | 716 (41.4%) | 164 (48.2%) | 425 (32.0%) |
| Other purpose | 73 (4.2%) | 48 (14.1%) | 185 (13.9%) |
| Intervention type, N | 1824 | 353 | 1393 |
| Drug | 1042 (57.1%) | 148 (41.9%) | 736 (52.8%) |
| Procedure | 24 (1.3%) | 15 (4.2%) | 103 (7.4%) |
| Biological | 712 (39.0%) | 103 (29.2%) | 208 (14.9%) |
| Behavioral | 8 (0.4%) | 93 (26.3%) | 115 (8.3%) |
| Device | 38 (2.1%) | 3 (0.8%) | 57 (4.1%) |
| Other intervention | 105 (5.8%) | 51 (14.4%) | 311 (22.3%) |
| Vaccine | 670 (36.7%) | 102 (28.9%) | 194 (13.9%) |
| Lead sponsor, N | 1824 | 353 | 1393 |
| Industry | 1542 (84.5%) | 2 (0.6%) | 6 (0.4%) |
| NIH | 0 (0.0%) | 211 (59.8%) | 0 (0.0%) |
| U.S. federal | 5 (0.3%) | 1 (0.3%) | 64 (4.6%) |
| Govt.- non-U.S. | 23 (1.3%) | 0 (0.0%) | 77 (5.5%) |
| Acad./hosp. | 198 (10.9%) | 115 (32.6%) | 996 (71.5%) |
| Consortium | 14 (0.8%) | 16 (4.5%) | 83 (6.0%) |
| Other | 42 (2.3%) | 8 (2.3%) | 167 (12.0%) |
| Trial facility, N | 1579 | 335 | 1323 |
| Single facility | 692 (43.8%) | 186 (55.5%) | 1023 (77.3%) |
| Multiple facilities | 887 (56.2%) | 149 (44.5%) | 300 (22.7%) |
| Enrollment, N | 1804 | 353 | 1370 |
| Median (IQR) | 126 (40, 375) | 120 (50, 408) | 126 (45, 400) |
| Sex/age, N | 1824 | 353 | 1393 |
| Female | 80 (4.4%) | 42 (11.9%) | 106 (7.6%) |
| Male | 42 (2.3%) | 10 (2.8%) | 57 (4.1%) |
| Both | 1702 (93.3%) | 301 (85.3%) | 1230 (88.3%) |
| Restricted to children^a^ | 351 (19.2%) | 36 (10.2%) | 214 (15.4%) |
| Excludes elderly^b^ | 938 (51.4%) | 183 (51.8%) | 596 (42.8%) |
| Masking/blinding, N | 1815 | 347 | 1377 |
| Open | 969 (53.4%) | 181 (52.2%) | 818 (59.4%) |
| Single-blind | 135 (7.4%) | 36 (10.4%) | 146 (10.6%) |
| Double-blind | 711 (39.2%) | 130 (37.5%) | 413 (30.0%) |
| Allocation, N | 1800 | 341 | 1357 |
| Randomized | 1273 (70.7%) | 272 (79.8%) | 1024 (75.5%) |
| Nonrandomized | 527 (29.3%) | 69 (20.2%) | 333 (24.5%) |
| Number of arms, N | 1742 | 349 | 1366 |
| 1 | 395 (22.7%) | 80 (22.9%) | 289 (21.2%) |
| 2 | 764 (43.9%) | 166 (47.6%) | 808 (59.2%) |
| 3 | 269 (15.4%) | 46 (13.2%) | 159 (11.6%) |
| 4 | 155 (8.9%) | 31 (8.9%) | 71 (5.2%) |
| 5 or more | 159 (9.1%) | 26 (7.4%) | 39 (2.9%) |
| Comparator, N | 1667 | 310 | 1284 |
| Active | 676 (40.6%) | 123 (39.7%) | 648 (50.5%) |
| Placebo | 394 (23.6%) | 69 (22.3%) | 310 (24.1%) |
| Phase, N | 1824 | 353 | 1393 |
| Phase 0 | 0 (0.0%) | 5 (1.4%) | 11 (0.8%) |
| Phase 1 | 320 (17.5%) | 91 (25.8%) | 120 (8.6%) |
| Phase 1/Phase 2 | 71 (3.9%) | 28 (7.9%) | 46 (3.3%) |
| Phase 2 | 514 (28.2%) | 67 (19.0%) | 179 (12.8%) |
| Phase 2/Phase 3 | 36 (2.0%) | 10 (2.8%) | 52 (3.7%) |
| Phase 3 | 494 (27.1%) | 39 (11.0%) | 175 (12.6%) |
| Phase 4 | 301 (16.5%) | 24 (6.8%) | 328 (23.5%) |
| N/A | 88 (4.8%) | 89 (25.2%) | 482 (34.6%) |
| Overall status, N | 1824 | 353 | 1393 |
| Not yet recruiting | 138 (7.6%) | 53 (15.0%) | 197 (14.1%) |
| Recruiting | 468 (25.7%) | 173 (49.0%) | 654 (46.9%) |
| Active, not recruiting | 326 (17.9%) | 55 (15.6%) | 174 (12.5%) |
| Completed | 829 (45.4%) | 64 (18.1%) | 331 (23.8%) |
| Terminated | 63 (3.5%) | 8 (2.3%) | 37 (2.7%) |
| DMC, N | 1824 | 353 | 1393 |
| Has DMC | 479 (26.3%) | 145 (41.1%) | 632 (45.4%) |
| No DMC | 729 (40.0%) | 94 (26.6%) | 694 (49.8%) |
| DMC missing | 616 (33.8%) | 114 (32.3%) | 67 (4.8%) |
| Regional distribution^c^, N | 1824 | 353 | 1393 |
| Africa | 86 (2.7%) | 36 (2.2%) | 175 (5.4%) |
| Central America | 92 (5.8%) | 15 (4.5%) | 5 (0.4%) |
| East Asia | 171 (10.8%) | 5 (1.5%) | 120 (9.1%) |
| Europe | 554 (35.1%) | 4 (1.2%) | 382 (28.9%) |
| Middle East | 48 (3.0%) | 1 (0.3%) | 44 (3.3%) |
| North America | 783 (49.6%) | 282 (84.2%) | 413 (31.2%) |
| North Asia | 41 (2.6%) | 3 (0.9%) | 5 (0.4%) |
| Pacifica | 96 (6.1%) | 1 (0.3%) | 11 (0.8%) |
| South America | 100 (6.3%) | 14 (4.2%) | 75 (5.7%) |
| South Asia | 52 (3.3%) | 8 (2.4%) | 64 (4.8%) |
| Southeast Asia | 77 (4.9%) | 9 (2.7%) | 58 (4.4%) |
| Unknown | 245 (13.4%) | 18 (5.1%) | 70 (5.0%) |
| The denominator for each variable is the number of trials reporting such data. "Other Purpose" includes "Diagnostic," "Supportive Care," "Screening," "Health Services Research," and "Basic Science." For "Intervention Type," the numerator is the number of trials with at least 1 intervention of this type. A study with multiple interventions may be represented in more than 1 intervention type; hence cumulative percentage will exceed 100%. "Other Intervention" includes "Radiation," "Dietary Supplement," and "Genetic." The "Comparator" variable identifies the number of trials designating that particular comparator. Since a study may have both a placebo and an active comparator arm, the cumulative percentage may exceed 100%. The "Recruiting" variable under "Overall Status" includes trials recruiting by invitation. "Terminated" includes trials that have been terminated, suspended, or withdrawn. The numerator in the "Regional Distribution" variable is the number of trials with at least one study site in that respective region. A multisite study may be represented in more than 1 region; hence, the cumulative percentage will exceed 100%. Abbreviations: DMC, data monitoring committee; IQR, interquartile range; NIH, US National Institutes of Health.  ^a^Children defined as ≤18 years of age. ^b^Elderly defined as >65 years of age. ^c^Individual countries by region are available at: http://www.clinicaltrials.gov/ct2/search/browse?brwse=locn_cat. | | | |
