# Supplementary material for: The State of Infectious Diseases Clinical Trials: A Systematic Review of ClinicalTrials.gov
Source: PLoS One. 2013 Oct 16;8(10):e77086. doi: 10.1371/journal.pone.0077086 (PMC3797691; doi:10.1371/journal.pone.0077086)
Supplement: Appendix S1 — List of ID Terms Used to Identify Potential Infectious Disease Trials from the ClinicalTrials.gov Registry. (DOC) [file pone.0077086.s005.doc]

| **Appendix S1.** List of ID Terms Used to Identify Potential Infectious Disease Trials from the ClinicalTrials.gov Registry. | | |
| --- | --- | --- |
| **MeSH Condition Terms from 2010 MeSH Thesaurus** | **Free-Text Conditions Appearing in ≥5 Studies** | **Intervention Terms Appearing in ≥4 Studies** |
| ABDOMINAL ABSCESS | ACQUIRED IMMUNE DEFICIENCY SYNDROME | ACYCLOVIR |
| ABORTION, SEPTIC | ACUTE HIV INFECTION | ALBENDAZOLE |
| ABSCESS | ACUTE OTITIS MEDIA | AMANTADINE |
| ACALCULOUS CHOLECYSTITIS | ACUTE RESPIRATORY INFECTION | AMIKACIN |
| ACANTHAMOEBA KERATITIS | AIDS | AMOXICILLIN |
| ACINETOBACTER INFECTIONS | ANTIBIOTIC-ASSOCIATED DIARRHEA | AMOXICILLIN-POTASSIUM CLAVULANATE COMBINATION |
| ACQUIRED IMMUNODEFICIENCY SYNDROME | BACTERIAL CONJUNCTIVITIS | AMPHOTERICIN B |
| ACTINOBACILLOSIS | BACTERIAL INFECTION | AMPICILLIN |
| ACTINOBACILLUS INFECTIONS | BACTERIAL VAGINOSIS | ANTHELMINTICS |
| ACTINOMYCETALES INFECTIONS | CANDIDEMIA | ANTI-BACTERIAL AGENTS |
| ACTINOMYCOSIS | CHRONIC HEPATITIS B | ANTI-HIV AGENTS |
| ACTINOMYCOSIS, CERVICOFACIAL | CHRONIC HEPATITIS C | ANTI-INFECTIVE AGENTS |
| ADENOPHOREA INFECTIONS | CHRONIC HEPATITIS C INFECTION | ANTI-INFECTIVE AGENTS, LOCAL |
| ADENOVIRIDAE INFECTIONS | CHRONIC HEPATITIS C VIRUS INFECTION | ANTI-RETROVIRAL AGENTS |
| ADENOVIRUS INFECTIONS, HUMAN | COMMUNITY ACQUIRED PNEUMONIA | ANTIFUNGAL AGENTS |
| AEROBIOSIS | COMMUNITY-ACQUIRED PNEUMONIA | ANTIMALARIALS |
| AFRICAN HORSE SICKNESS | CRYPTOCOCCAL MENINGITIS | ANTIMONY SODIUM GLUCONATE |
| AFRICAN SWINE FEVER | CUTANEOUS LEISHMANIASIS | ANTIPARASITIC AGENTS |
| AIDS ARTERITIS, CENTRAL NERVOUS SYSTEM | CYTOMEGALOVIRUS INFECTION | ANTIPROTOZOAL AGENTS |
| AIDS DEMENTIA COMPLEX | DENGUE FEVER | ANTITUBERCULAR AGENTS |
| AIDS SERODIAGNOSIS | DIABETIC FOOT ULCER | ANTIVIRAL AGENTS |
| AIDS-ASSOCIATED NEPHROPATHY | DIABETIC FOOT ULCERS | ARTEMISININS |
| AIDS-RELATED COMPLEX | DIARRHOEA | ATOVAQUONE |
| AIDS-RELATED OPPORTUNISTIC INFECTIONS | FALCIPARUM MALARIA | AZITHROMYCIN |
| ALEUTIAN MINK DISEASE | FOOT ULCER, DIABETIC | AZTREONAM |
| ALPHAVIRUS INFECTIONS | FUNGAL INFECTION | BACITRACIN |
| AMEBIASIS | GENITAL WARTS | BCG VACCINE |
| AMPICILLIN RESISTANCE | H1N1 INFLUENZA VIRUS | BOTULINUM ANTITOXIN |
| ANAEROBIOSIS | HAEMOPHILUS INFLUENZAE INFECTIONS | CD4 IMMUNOADHESINS |
| ANAPLASMATACEAE INFECTIONS | HAEMOPHILUS INFLUENZAE TYPE B | CEFAZOLIN |
| ANAPLASMOSIS | HAEMOPHILUS INFLUENZAE TYPE B DISEASE | CEFIXIME |
| ANCYLOSTOMIASIS | HCV | CEFOXITIN |
| ANEURYSM, INFECTED | HCV INFECTION | CEFTAZIDIME |
| ANGIOMATOSIS, BACILLARY | HEAD LICE | CEFTRIAXONE |
| ANISAKIASIS | HELICOBACTER PYLORI INFECTION | CEFUROXIME |
| ANTHRAX | HEPATIC CIRRHOSIS | CEPHALEXIN |
| ANTIBIOSIS | HEPATITIS B VIRUS | CEPHALOSPORINS |
| ANTIBIOTIC PROPHYLAXIS | HEPATITIS C INFECTION | CHLOROQUINE |
| ANTIBODY-COATED BACTERIA TEST, URINARY | HEPATITIS C VIRUS | CILASTATIN |
| ANTIRETROVIRAL THERAPY, HIGHLY ACTIVE | HEPATITIS C VIRUS INFECTION | CIPROFLOXACIN |
| APPENDICITIS | HIV INFECTION | CLARITHROMYCIN |
| ARBOVIRUS INFECTIONS | HIV PREVENTION | CLAVULANIC ACIDS |
| ARENAVIRIDAE INFECTIONS | HIV-1 INFECTION | CLINDAMYCIN |
| ARTERIVIRUS INFECTIONS | HIV-1 INFECTIONS | CLOTRIMAZOLE |
| ARTHRITIS, INFECTIOUS | HIV/AIDS | CLOXACILLIN |
| ASCARIASIS | HPV | COLISTIN |
| ASCARIDIASIS | HPV INFECTIONS | DACTINOMYCIN |
| ASCARIDIDA INFECTIONS | HUMAN IMMUNODEFICIENCY VIRUS | DAPSONE |
| ASPERGILLOSIS | HUMAN IMMUNODEFICIENCY VIRUS (HIV) | DAPTOMYCIN |
| ASPERGILLOSIS, ALLERGIC BRONCHOPULMONARY | HUMAN PAPILLOMAVIRUS INFECTION | DELAVIRDINE |
| ASTROVIRIDAE INFECTIONS | INFECTION, HUMAN IMMUNODEFICIENCY VIRUS | DIDANOSINE |
| AUTOTROPHIC PROCESSES | INFECTIONS | DOXYCYCLINE |
| AVIAN LEUKOSIS | INFECTIOUS DISEASES | ECHINOCANDINS |
| AVULAVIRUS INFECTIONS | INFECTIVE ENDOCARDITIS | ERYTHROMYCIN |
| BABESIOSIS | INFLUENZA | ERYTHROMYCIN ESTOLATE |
| BACILLACEAE INFECTIONS | INFLUENZA DISEASE | ERYTHROMYCIN ETHYLSUCCINATE |
| BACTEREMIA | INFLUENZA INFECTION | ETHAMBUTOL |
| BACTERIAL ADHESION | INVASIVE CANDIDIASIS | FLOXACILLIN |
| BACTERIAL INFECTIONS | JAPANESE ENCEPHALITIS | FLUCONAZOLE |
| BACTERIAL INFECTIONS AND MYCOSES | LATENT TUBERCULOSIS INFECTION | FLUCYTOSINE |
| BACTERIAL PHYSIOLOGICAL PHENOMENA | MALARIA IN PREGNANCY | FLUOROQUINOLONES |
| BACTERIAL PROCESSES | MENINGOCOCCAL DISEASE | FOSCARNET |
| BACTERIAL SHEDDING | MENINGOCOCCAL INFECTION | FOSFOMYCIN |
| BACTERIAL TRANSLOCATION | MENINGOCOCCAL MENINGITIS | FUSIDIC ACID |
| BACTERIAL TYPING TECHNIQUES | MENINGOCOCCEMIA | GANCICLOVIR |
| BACTERIOCIN PLASMIDS | METHICILLIN-RESISTANT STAPHYLOCOCCUS AUREUS | GENTAMICINS |
| BACTERIOLOGICAL TECHNIQUES | MRSA INFECTION | HEPATITIS B ANTIBODIES |
| BACTERIOLYSIS | MYXOVIRUS INFECTION | HIV ANTIBODIES |
| BACTERIOPHAGE TYPING | NECROTIZING ENTEROCOLITIS | HIV FUSION INHIBITORS |
| BACTERIURIA | NEONATAL SEPSIS | HIV INTEGRASE INHIBITORS |
| BACTEROIDACEAE INFECTIONS | OTITIS MEDIA WITH EFFUSION | HIV PROTEASE INHIBITORS |
| BACTEROIDES INFECTIONS | PANDEMIC INFLUENZA | IMIPENEM |
| BALANITIS | PEDICULOSIS | INDINAVIR |
| BALANTIDIASIS | PERTUSSIS | INTEGRASE INHIBITORS |
| BARTONELLA INFECTIONS | PLASMODIUM FALCIPARUM MALARIA | INTERFERON ALFA-2A |
| BARTONELLACEAE INFECTIONS | PNEUMOCOCCAL DISEASE | ISONIAZID |
| BELL PALSY | PNEUMOCOCCAL DISEASES | ITRACONAZOLE |
| BETA-LACTAM RESISTANCE | PSEUDOMONAS AERUGINOSA | IVERMECTIN |
| BIFIDOBACTERIALES INFECTIONS | PULMONARY TUBERCULOSIS | KETOCONAZOLE |
| BIOFILMS | RESPIRATORY INFECTION | LAMIVUDINE |
| BIRNAVIRIDAE INFECTIONS | RHINOCONJUNCTIVITIS | LEVAMISOLE |
| BLACKWATER FEVER | RHINOSINUSITIS | MEBENDAZOLE |
| BLASTOCYSTIS INFECTIONS | ROTAVIRUS GASTROENTERITIS | MEFLOQUINE |
| BLASTOMYCOSIS | SEASONAL INFLUENZA | METHICILLIN |
| BLEPHARITIS | SEPTIC SHOCK | METRONIDAZOLE |
| BLOOD BACTERICIDAL ACTIVITY | SEVERE SEPSIS | MICONAZOLE |
| BLUETONGUE | SEXUALLY TRANSMITTED INFECTIONS | MILRINONE |
| BONE DISEASES, INFECTIOUS | SHINGLES | MINOCYCLINE |
| BORDER DISEASE | STAPHYLOCOCCUS AUREUS | MUPIROCIN |
| BORDETELLA INFECTIONS | SURGICAL SITE INFECTION | NELFINAVIR |
| BORNA DISEASE | SWINE-ORIGIN A/H1N1 INFLUENZA | NEOMYCIN |
| BORRELIA INFECTIONS | TRAVELERS' DIARRHEA | NEVIRAPINE |
| BOTULISM | UNCOMPLICATED MALARIA | NITROFURANTOIN |
| BOUTONNEUSE FEVER | UPPER RESPIRATORY TRACT INFECTION | NONOXYNOL |
| BOVINE VIRUS DIARRHEA-MUCOSAL DISEASE | UPPER RESPIRATORY TRACT INFECTIONS | NORFLOXACIN |
| BRAIN ABSCESS | URINARY TRACT INFECTION | NYSTATIN |
| BRONCHIOLITIS, VIRAL | VACCINES, PNEUMOCOCCAL CONJUGATE VACCINE | OFLOXACIN |
| BRONCHOPNEUMONIA | VARICELLA | OSELTAMIVIR |
| BRUCELLOSIS | VENTILATOR ASSOCIATED PNEUMONIA | OXACILLIN |
| BRUCELLOSIS, BOVINE | VISCERAL LEISHMANIASIS | PAROMOMYCIN |
| BUNYAVIRIDAE INFECTIONS |  | PENICILLIN G |
| BURKHOLDERIA INFECTIONS |  | PENICILLIN V |
| BURULI ULCER |  | PENTAMIDINE |
| CALICIVIRIDAE INFECTIONS |  | PERMETHRIN |
| CAMPYLOBACTER INFECTIONS |  | PIPERACILLIN |
| CANDIDIASIS |  | POLYMYXIN B |
| CANDIDIASIS, CHRONIC MUCOCUTANEOUS |  | POLYMYXINS |
| CANDIDIASIS, CUTANEOUS |  | POVIDONE |
| CANDIDIASIS, ORAL |  | PRAZIQUANTEL |
| CANDIDIASIS, VULVOVAGINAL |  | PRIMAQUINE |
| CARBUNCLE |  | PROBENECID |
| CARDIOVASCULAR INFECTIONS |  | PROTEASE INHIBITORS |
| CARDIOVIRUS INFECTIONS |  | PYRAZINAMIDE |
| CAT-SCRATCH DISEASE |  | QUINACRINE |
| CATHETER-RELATED INFECTIONS |  | QUINIDINE |
| CD4 LYMPHOCYTE COUNT |  | QUININE |
| CD4-CD8 RATIO |  | REVERSE TRANSCRIPTASE INHIBITORS |
| CELL TRANSFORMATION, VIRAL |  | RIBAVIRIN |
| CELLULITIS |  | RIFABUTIN |
| CENTRAL NERVOUS SYSTEM BACTERIAL INFECTIONS |  | RIFAMPIN |
| CENTRAL NERVOUS SYSTEM FUNGAL INFECTIONS |  | RITONAVIR |
| CENTRAL NERVOUS SYSTEM HELMINTHIASIS |  | SAQUINAVIR |
| CENTRAL NERVOUS SYSTEM INFECTIONS |  | SPIRAMYCIN |
| CENTRAL NERVOUS SYSTEM PARASITIC INFECTIONS |  | STAVUDINE |
| CENTRAL NERVOUS SYSTEM PROTOZOAL INFECTIONS |  | STREPTOMYCIN |
| CENTRAL NERVOUS SYSTEM VIRAL DISEASES |  | SULBACTAM |
| CEPHALOSPORIN RESISTANCE |  | SULFAMETHOXAZOLE |
| CESTODE INFECTIONS |  | SURAMIN |
| CHAGAS DISEASE |  | TETRACYCLINE |
| CHANCRE |  | TINIDAZOLE |
| CHANCROID |  | TOBRAMYCIN |
| CHEMOAUTOTROPHIC GROWTH |  | TRIMETHOPRIM |
| CHICKENPOX |  | TRIMETHOPRIM-SULFAMETHOXAZOLE COMBINATION |
| CHLAMYDIA INFECTIONS |  | VANCOMYCIN |
| CHLAMYDIACEAE INFECTIONS |  | ZANAMIVIR |
| CHLAMYDOPHILA INFECTIONS |  | ZIDOVUDINE |
| CHLORAMPHENICOL RESISTANCE |  |  |
| CHOLECYSTITIS |  |  |
| CHOLECYSTITIS, ACUTE |  |  |
| CHOLERA |  |  |
| CHOLERA MORBUS |  |  |
| CHORIOAMNIONITIS |  |  |
| CHORIORETINITIS |  |  |
| CHROMOBLASTOMYCOSIS |  |  |
| CHROMOSOMES, ARTIFICIAL, BACTERIAL |  |  |
| CHROMOSOMES, ARTIFICIAL, P1 BACTERIOPHAGE |  |  |
| CHROMOSOMES, ARTIFICIAL, YEAST |  |  |
| CHROMOSOMES, BACTERIAL |  |  |
| CHROMOSOMES, FUNGAL |  |  |
| CIGUATERA POISONING |  |  |
| CILIOPHORA INFECTIONS |  |  |
| CIRCOVIRIDAE INFECTIONS |  |  |
| CLASSICAL SWINE FEVER |  |  |
| CLONORCHIASIS |  |  |
| CLOSTRIDIUM INFECTIONS |  |  |
| COCCIDIOIDOMYCOSIS |  |  |
| COCCIDIOSIS |  |  |
| COLONY COUNT, MICROBIAL |  |  |
| COLONY-FORMING UNITS ASSAY |  |  |
| COLORADO TICK FEVER |  |  |
| COMMON COLD |  |  |
| COMMUNICABLE DISEASES |  |  |
| COMMUNICABLE DISEASES, EMERGING |  |  |
| COMMUNITY-ACQUIRED INFECTIONS |  |  |
| CONDYLOMATA ACUMINATA |  |  |
| CONJUNCTIVITIS, ACUTE HEMORRHAGIC |  |  |
| CONJUNCTIVITIS, BACTERIAL |  |  |
| CONJUNCTIVITIS, INCLUSION |  |  |
| CONJUNCTIVITIS, VIRAL |  |  |
| CONTACT TRACING |  |  |
| CORONAVIRIDAE INFECTIONS |  |  |
| CORONAVIRUS INFECTIONS |  |  |
| CORYNEBACTERIUM INFECTIONS |  |  |
| COWPOX |  |  |
| COXSACKIEVIRUS INFECTIONS |  |  |
| CREUTZFELDT-JAKOB SYNDROME |  |  |
| CROSS INFECTION |  |  |
| CROUP |  |  |
| CRYPTOCOCCOSIS |  |  |
| CRYPTOSPORIDIOSIS |  |  |
| CULTURE TECHNIQUES |  |  |
| CYCLOSPORIASIS |  |  |
| CYSTICERCOSIS |  |  |
| CYTOMEGALOVIRUS INFECTIONS |  |  |
| CYTOMEGALOVIRUS RETINITIS |  |  |
| CYTOPATHOGENIC EFFECT, VIRAL |  |  |
| DACRYOCYSTITIS |  |  |
| DELTARETROVIRUS INFECTIONS |  |  |
| DENGUE |  |  |
| DENGUE HEMORRHAGIC FEVER |  |  |
| DERMATOMYCOSES |  |  |
| DESULFOVIBRIONACEAE INFECTIONS |  |  |
| DIARRHEA |  |  |
| DICROCOELIASIS |  |  |
| DICTYOCAULUS INFECTIONS |  |  |
| DIENTAMOEBIASIS |  |  |
| DIPETALONEMA INFECTIONS |  |  |
| DIPHTHERIA |  |  |
| DIPHYLLOBOTHRIASIS |  |  |
| DIRECTLY OBSERVED THERAPY |  |  |
| DIROFILARIASIS |  |  |
| DISCITIS |  |  |
| DISEASE NOTIFICATION |  |  |
| DISK DIFFUSION ANTIMICROBIAL TESTS |  |  |
| DISTEMPER |  |  |
| DIVERTICULITIS |  |  |
| DIVERTICULITIS, COLONIC |  |  |
| DNA VIRUS INFECTIONS |  |  |
| DOURINE |  |  |
| DRACUNCULIASIS |  |  |
| DRUG RESISTANCE, BACTERIAL |  |  |
| DRUG RESISTANCE, FUNGAL |  |  |
| DRUG RESISTANCE, MICROBIAL |  |  |
| DRUG RESISTANCE, MULTIPLE, BACTERIAL |  |  |
| DRUG RESISTANCE, MULTIPLE, FUNGAL |  |  |
| DRUG RESISTANCE, MULTIPLE, VIRAL |  |  |
| DRUG RESISTANCE, VIRAL |  |  |
| DYSENTERY |  |  |
| DYSENTERY, AMEBIC |  |  |
| DYSENTERY, BACILLARY |  |  |
| ECHINOCOCCOSIS |  |  |
| ECHINOCOCCOSIS, HEPATIC |  |  |
| ECHINOCOCCOSIS, PULMONARY |  |  |
| ECHINOSTOMIASIS |  |  |
| ECHOVIRUS INFECTIONS |  |  |
| ECTHYMA |  |  |
| ECTHYMA, CONTAGIOUS |  |  |
| ECTOPARASITIC INFESTATIONS |  |  |
| ECTROMELIA, INFECTIOUS |  |  |
| EDEMA DISEASE OF SWINE |  |  |
| EHRLICHIOSIS |  |  |
| ELEPHANTIASIS |  |  |
| ELEPHANTIASIS, FILARIAL |  |  |
| EMPHYSEMATOUS CHOLECYSTITIS |  |  |
| EMPYEMA |  |  |
| EMPYEMA, PLEURAL |  |  |
| EMPYEMA, SUBDURAL |  |  |
| EMPYEMA, TUBERCULOUS |  |  |
| ENCEPHALITIS |  |  |
| ENCEPHALITIS, ARBOVIRUS |  |  |
| ENCEPHALITIS, CALIFORNIA |  |  |
| ENCEPHALITIS, HERPES SIMPLEX |  |  |
| ENCEPHALITIS, JAPANESE |  |  |
| ENCEPHALITIS, ST. LOUIS |  |  |
| ENCEPHALITIS, TICK-BORNE |  |  |
| ENCEPHALITIS, VARICELLA ZOSTER |  |  |
| ENCEPHALITIS, VIRAL |  |  |
| ENCEPHALITOZOONOSIS |  |  |
| ENCEPHALOMYELITIS |  |  |
| ENCEPHALOMYELITIS, EASTERN EQUINE |  |  |
| ENCEPHALOMYELITIS, ENZOOTIC PORCINE |  |  |
| ENCEPHALOMYELITIS, EQUINE |  |  |
| ENCEPHALOMYELITIS, VENEZUELAN EQUINE |  |  |
| ENCEPHALOMYELITIS, WESTERN EQUINE |  |  |
| ENCEPHALOPATHY, BOVINE SPONGIFORM |  |  |
| ENDOCARDITIS |  |  |
| ENDOCARDITIS, BACTERIAL |  |  |
| ENDOCARDITIS, SUBACUTE BACTERIAL |  |  |
| ENDOGENOUS RETROVIRUSES |  |  |
| ENDOMETRITIS |  |  |
| ENDOPHTHALMITIS |  |  |
| ENDOTOXEMIA |  |  |
| ENOPLIDA INFECTIONS |  |  |
| ENTAMOEBIASIS |  |  |
| ENTERITIS, TRANSMISSIBLE, OF TURKEYS |  |  |
| ENTEROBACTERIACEAE INFECTIONS |  |  |
| ENTEROBIASIS |  |  |
| ENTEROCOLITIS, NECROTIZING |  |  |
| ENTEROCOLITIS, PSEUDOMEMBRANOUS |  |  |
| ENTEROTOXEMIA |  |  |
| ENTEROVIRUS INFECTIONS |  |  |
| ENZOOTIC BOVINE LEUKOSIS |  |  |
| EPHEMERAL FEVER |  |  |
| EPIDERMITIS, EXUDATIVE, OF SWINE |  |  |
| EPIDERMODYSPLASIA VERRUCIFORMIS |  |  |
| EPIDIDYMITIS |  |  |
| EPIDURAL ABSCESS |  |  |
| EPIGLOTTITIS |  |  |
| EPSTEIN-BARR VIRUS INFECTIONS |  |  |
| EQUINE INFECTIOUS ANEMIA |  |  |
| ERYSIPELAS |  |  |
| ERYSIPELOID |  |  |
| ERYSIPELOTHRIX INFECTIONS |  |  |
| ERYTHEMA CHRONICUM MIGRANS |  |  |
| ERYTHEMA INDURATUM |  |  |
| ERYTHEMA INFECTIOSUM |  |  |
| ERYTHRASMA |  |  |
| ESCHERICHIA COLI INFECTIONS |  |  |
| ETHMOID SINUSITIS |  |  |
| EUGLENOZOA INFECTIONS |  |  |
| EXANTHEMA SUBITUM |  |  |
| EXTENSIVELY DRUG-RESISTANT TUBERCULOSIS |  |  |
| EYE INFECTIONS |  |  |
| EYE INFECTIONS, BACTERIAL |  |  |
| EYE INFECTIONS, FUNGAL |  |  |
| EYE INFECTIONS, PARASITIC |  |  |
| EYE INFECTIONS, VIRAL |  |  |
| FASCIITIS, NECROTIZING |  |  |
| FASCIOLIASIS |  |  |
| FASCIOLOIDIASIS |  |  |
| FATIGUE SYNDROME, CHRONIC |  |  |
| FELINE ACQUIRED IMMUNODEFICIENCY SYNDROME |  |  |
| FELINE INFECTIOUS PERITONITIS |  |  |
| FELINE PANLEUKOPENIA |  |  |
| FEVER |  |  |
| FEVER OF UNKNOWN ORIGIN |  |  |
| FILARIASIS |  |  |
| FILOVIRIDAE INFECTIONS |  |  |
| FLAVIVIRIDAE INFECTIONS |  |  |
| FLAVIVIRUS INFECTIONS |  |  |
| FLAVOBACTERIACEAE INFECTIONS |  |  |
| FLEXIBACTERACEAE INFECTIONS |  |  |
| FLUORESCENT TREPONEMAL ANTIBODY-ABSORPTION TEST |  |  |
| FOCAL INFECTION |  |  |
| FOLLICULITIS |  |  |
| FOODBORNE DISEASES |  |  |
| FOOT ROT |  |  |
| FOOT-AND-MOUTH DISEASE |  |  |
| FOURNIER GANGRENE |  |  |
| FOWLPOX |  |  |
| FRONTAL SINUSITIS |  |  |
| FUNGEMIA |  |  |
| FURUNCULOSIS |  |  |
| FUSOBACTERIACEAE INFECTIONS |  |  |
| FUSOBACTERIUM INFECTIONS |  |  |
| GANGRENE |  |  |
| GAS GANGRENE |  |  |
| GASTROENTERITIS |  |  |
| GASTROENTERITIS, TRANSMISSIBLE, OF SWINE |  |  |
| GENE EXPRESSION REGULATION, BACTERIAL |  |  |
| GENE EXPRESSION REGULATION, FUNGAL |  |  |
| GENE EXPRESSION REGULATION, VIRAL |  |  |
| GENES, ARCHAEAL |  |  |
| GENES, BACTERIAL |  |  |
| GENES, ENV |  |  |
| GENES, FUNGAL |  |  |
| GENES, GAG |  |  |
| GENES, HELMINTH |  |  |
| GENES, IMMEDIATE-EARLY |  |  |
| GENES, INTRACISTERNAL A-PARTICLE |  |  |
| GENES, MATING TYPE, FUNGAL |  |  |
| GENES, MDR |  |  |
| GENES, NEF |  |  |
| GENES, POL |  |  |
| GENES, PROTOZOAN |  |  |
| GENES, PX |  |  |
| GENES, REV |  |  |
| GENES, TAT |  |  |
| GENES, VIF |  |  |
| GENES, VIRAL |  |  |
| GENES, VPR |  |  |
| GENES, VPU |  |  |
| GENOME, ARCHAEAL |  |  |
| GENOME, BACTERIAL |  |  |
| GENOME, FUNGAL |  |  |
| GENOME, PROTOZOAN |  |  |
| GENOME, VIRAL |  |  |
| GEOTRICHOSIS |  |  |
| GERSTMANN-STRAUSSLER-SCHEINKER DISEASE |  |  |
| GIARDIASIS |  |  |
| GLANDERS |  |  |
| GLOSSITIS |  |  |
| GONORRHEA |  |  |
| GRAM-NEGATIVE BACTERIAL INFECTIONS |  |  |
| GRAM-POSITIVE BACTERIAL INFECTIONS |  |  |
| GRANULOMA INGUINALE |  |  |
| GRANULOMA, PYOGENIC |  |  |
| HAEMONCHIASIS |  |  |
| HAEMOPHILUS INFECTIONS |  |  |
| HAND, FOOT AND MOUTH DISEASE |  |  |
| HANTAVIRUS INFECTIONS |  |  |
| HANTAVIRUS PULMONARY SYNDROME |  |  |
| HEARTWATER DISEASE |  |  |
| HELICOBACTER INFECTIONS |  |  |
| HELMINTHIASIS |  |  |
| HELMINTHIASIS, ANIMAL |  |  |
| HEMAGGLUTINATION, VIRAL |  |  |
| HEMORRHAGIC FEVER WITH RENAL SYNDROME |  |  |
| HEMORRHAGIC FEVER, AMERICAN |  |  |
| HEMORRHAGIC FEVER, CRIMEAN |  |  |
| HEMORRHAGIC FEVER, EBOLA |  |  |
| HEMORRHAGIC FEVER, OMSK |  |  |
| HEMORRHAGIC FEVERS, VIRAL |  |  |
| HEMORRHAGIC SEPTICEMIA |  |  |
| HEMORRHAGIC SEPTICEMIA, VIRAL |  |  |
| HEMORRHAGIC SYNDROME, BOVINE |  |  |
| HENIPAVIRUS INFECTIONS |  |  |
| HEPADNAVIRIDAE INFECTIONS |  |  |
| HEPATITIS A |  |  |
| HEPATITIS B |  |  |
| HEPATITIS B, CHRONIC |  |  |
| HEPATITIS C |  |  |
| HEPATITIS C, CHRONIC |  |  |
| HEPATITIS D |  |  |
| HEPATITIS D, CHRONIC |  |  |
| HEPATITIS E |  |  |
| HEPATITIS, ANIMAL |  |  |
| HEPATITIS, CHRONIC |  |  |
| HEPATITIS, INFECTIOUS CANINE |  |  |
| HEPATITIS, VIRAL, ANIMAL |  |  |
| HEPATITIS, VIRAL, HUMAN |  |  |
| HERPANGINA |  |  |
| HERPES GENITALIS |  |  |
| HERPES LABIALIS |  |  |
| HERPES SIMPLEX |  |  |
| HERPES ZOSTER |  |  |
| HERPES ZOSTER OPHTHALMICUS |  |  |
| HERPES ZOSTER OTICUS |  |  |
| HERPESVIRIDAE INFECTIONS |  |  |
| HETEROTROPHIC PROCESSES |  |  |
| HIDRADENITIS |  |  |
| HIDRADENITIS SUPPURATIVA |  |  |
| HISTOPLASMOSIS |  |  |
| HIV ENHANCER |  |  |
| HIV ENTEROPATHY |  |  |
| HIV INFECTIONS |  |  |
| HIV LONG TERMINAL REPEAT |  |  |
| HIV SERONEGATIVITY |  |  |
| HIV SEROPOSITIVITY |  |  |
| HIV SEROPREVALENCE |  |  |
| HIV WASTING SYNDROME |  |  |
| HIV-ASSOCIATED LIPODYSTROPHY SYNDROME |  |  |
| HOOKWORM INFECTIONS |  |  |
| HORDEOLUM |  |  |
| HOST-PARASITE INTERACTIONS |  |  |
| HOST-PATHOGEN INTERACTIONS |  |  |
| HTLV-I INFECTIONS |  |  |
| HTLV-II INFECTIONS |  |  |
| HYMENOLEPIASIS |  |  |
| HYPODERMYIASIS |  |  |
| IMMUNE RECONSTITUTION INFLAMMATORY SYNDROME |  |  |
| IMMUNITY, HERD |  |  |
| IMMUNITY, INNATE |  |  |
| IMMUNITY, MATERNALLY-ACQUIRED |  |  |
| IMMUNITY, MUCOSAL |  |  |
| IMMUNIZATION |  |  |
| IMMUNIZATION SCHEDULE |  |  |
| IMMUNIZATION, PASSIVE |  |  |
| IMMUNIZATION, SECONDARY |  |  |
| IMMUNOCOMPROMISED HOST |  |  |
| IMPETIGO |  |  |
| INCLUSION BODIES, VIRAL |  |  |
| INFECTION |  |  |
| INFECTIOUS BOVINE RHINOTRACHEITIS |  |  |
| INFECTIOUS MONONUCLEOSIS |  |  |
| INFLUENZA IN BIRDS |  |  |
| INFLUENZA, HUMAN |  |  |
| INSECTICIDE RESISTANCE |  |  |
| INSECTICIDE-TREATED BEDNETS |  |  |
| INSOMNIA, FATAL FAMILIAL |  |  |
| INTERTRIGO |  |  |
| INTESTINAL DISEASES, PARASITIC |  |  |
| INVASIVE PULMONARY ASPERGILLOSIS |  |  |
| ISOSPORIASIS |  |  |
| KANAMYCIN RESISTANCE |  |  |
| KAPOSI VARICELLIFORM ERUPTION |  |  |
| KERATITIS |  |  |
| KERATITIS, DENDRITIC |  |  |
| KERATITIS, HERPETIC |  |  |
| KERATOCONJUNCTIVITIS, INFECTIOUS |  |  |
| KING'S EVIL |  |  |
| KLEBSIELLA INFECTIONS |  |  |
| KURU |  |  |
| KYASANUR FOREST DISEASE |  |  |
| LABORATORY INFECTION |  |  |
| LARVA MIGRANS |  |  |
| LARVA MIGRANS, VISCERAL |  |  |
| LASSA FEVER |  |  |
| LATENT TUBERCULOSIS |  |  |
| LEGIONELLOSIS |  |  |
| LEGIONNAIRES' DISEASE |  |  |
| LEISHMANIASIS |  |  |
| LEISHMANIASIS, CUTANEOUS |  |  |
| LEISHMANIASIS, DIFFUSE CUTANEOUS |  |  |
| LEISHMANIASIS, MUCOCUTANEOUS |  |  |
| LEISHMANIASIS, VISCERAL |  |  |
| LENTIVIRUS INFECTIONS |  |  |
| LEPROSY |  |  |
| LEPROSY, BORDERLINE |  |  |
| LEPROSY, LEPROMATOUS |  |  |
| LEPROSY, MULTIBACILLARY |  |  |
| LEPROSY, PAUCIBACILLARY |  |  |
| LEPROSY, TUBERCULOID |  |  |
| LEPTOSPIROSIS |  |  |
| LEUKOENCEPHALOPATHY, PROGRESSIVE MULTIFOCAL |  |  |
| LEUKOPLAKIA, HAIRY |  |  |
| LICE INFESTATIONS |  |  |
| LIMULUS TEST |  |  |
| LISTERIA INFECTIONS |  |  |
| LIVER ABSCESS |  |  |
| LIVER ABSCESS, AMEBIC |  |  |
| LIVER ABSCESS, PYOGENIC |  |  |
| LIVER DISEASES, PARASITIC |  |  |
| LOIASIS |  |  |
| LOUPING ILL |  |  |
| LUDWIG'S ANGINA |  |  |
| LUMPY SKIN DISEASE |  |  |
| LUNG ABSCESS |  |  |
| LUNG DISEASES, FUNGAL |  |  |
| LUNG DISEASES, PARASITIC |  |  |
| LUPUS VULGARIS |  |  |
| LYME DISEASE |  |  |
| LYME NEUROBORRELIOSIS |  |  |
| LYMPHADENITIS |  |  |
| LYMPHANGITIS |  |  |
| LYMPHOCYTIC CHORIOMENINGITIS |  |  |
| LYMPHOGRANULOMA VENEREUM |  |  |
| LYMPHOMA, AIDS-RELATED |  |  |
| LYMPHOMA, PRIMARY EFFUSION |  |  |
| MALARIA |  |  |
| MALARIA, AVIAN |  |  |
| MALARIA, CEREBRAL |  |  |
| MALARIA, FALCIPARUM |  |  |
| MALARIA, VIVAX |  |  |
| MALIGNANT CATARRH |  |  |
| MANDATORY REPORTING |  |  |
| MANSONELLIASIS |  |  |
| MARBURG VIRUS DISEASE |  |  |
| MAREK DISEASE |  |  |
| MASS VACCINATION |  |  |
| MASTITIS |  |  |
| MASTITIS, BOVINE |  |  |
| MASTOIDITIS |  |  |
| MAXILLARY SINUSITIS |  |  |
| MEASLES |  |  |
| MEDIASTINITIS |  |  |
| MEGACOLON, TOXIC |  |  |
| MELIOIDOSIS |  |  |
| MENINGITIS |  |  |
| MENINGITIS, ASEPTIC |  |  |
| MENINGITIS, BACTERIAL |  |  |
| MENINGITIS, CRYPTOCOCCAL |  |  |
| MENINGITIS, ESCHERICHIA COLI |  |  |
| MENINGITIS, FUNGAL |  |  |
| MENINGITIS, HAEMOPHILUS |  |  |
| MENINGITIS, LISTERIA |  |  |
| MENINGITIS, MENINGOCOCCAL |  |  |
| MENINGITIS, PNEUMOCOCCAL |  |  |
| MENINGITIS, VIRAL |  |  |
| MENINGOCOCCAL INFECTIONS |  |  |
| MENINGOENCEPHALITIS |  |  |
| MESOMYCETOZOEA INFECTIONS |  |  |
| METHICILLIN RESISTANCE |  |  |
| MICROBIAL INTERACTIONS |  |  |
| MICROBIAL SENSITIVITY TESTS |  |  |
| MICROBIOLOGICAL PHENOMENA |  |  |
| MICROBIOLOGICAL PROCESSES |  |  |
| MICROBIOLOGICAL TECHNIQUES |  |  |
| MICROSPORIDIOSIS |  |  |
| MINK VIRAL ENTERITIS |  |  |
| MITE INFESTATIONS |  |  |
| MOLLUSCUM CONTAGIOSUM |  |  |
| MONIEZIASIS |  |  |
| MONKEYPOX |  |  |
| MONONEGAVIRALES INFECTIONS |  |  |
| MORAXELLACEAE INFECTIONS |  |  |
| MORBILLIVIRUS INFECTIONS |  |  |
| MOSQUITO NETS |  |  |
| MUCORMYCOSIS |  |  |
| MUMPS |  |  |
| MURINE ACQUIRED IMMUNODEFICIENCY SYNDROME |  |  |
| MYCETOMA |  |  |
| MYCOBACTERIUM AVIUM-INTRACELLULARE INFECTION |  |  |
| MYCOBACTERIUM INFECTIONS |  |  |
| MYCOBACTERIUM INFECTIONS, ATYPICAL |  |  |
| MYCOLOGICAL TYPING TECHNIQUES |  |  |
| MYCOPLASMA INFECTIONS |  |  |
| MYCOPLASMATALES INFECTIONS |  |  |
| MYCOSES |  |  |
| MYIASIS |  |  |
| MYOCARDITIS |  |  |
| MYXOMATOSIS, INFECTIOUS |  |  |
| NAIROBI SHEEP DISEASE |  |  |
| NECATORIASIS |  |  |
| NEISSERIACEAE INFECTIONS |  |  |
| NEMATODE INFECTIONS |  |  |
| NEUROASPERGILLOSIS |  |  |
| NEUROCYSTICERCOSIS |  |  |
| NEUROSCHISTOSOMIASIS |  |  |
| NEUROSYPHILIS |  |  |
| NEWCASTLE DISEASE |  |  |
| NIDOVIRALES INFECTIONS |  |  |
| NOCARDIA INFECTIONS |  |  |
| OESOPHAGOSTOMIASIS |  |  |
| ONCHOCERCIASIS |  |  |
| ONCHOCERCIASIS, OCULAR |  |  |
| ONCOLYTIC VIROTHERAPY |  |  |
| ONYCHOMYCOSIS |  |  |
| OPHTHALMIA NEONATORUM |  |  |
| OPISTHORCHIASIS |  |  |
| OPPORTUNISTIC INFECTIONS |  |  |
| ORBITAL CELLULITIS |  |  |
| ORTHOMYXOVIRIDAE INFECTIONS |  |  |
| OSTEOMYELITIS |  |  |
| OSTERTAGIASIS |  |  |
| OTITIS |  |  |
| OTITIS EXTERNA |  |  |
| OTITIS MEDIA |  |  |
| OTITIS MEDIA WITH EFFUSION |  |  |
| OTITIS MEDIA, SUPPURATIVE |  |  |
| OXYURIASIS |  |  |
| OXYURIDA INFECTIONS |  |  |
| PAPILLOMAVIRUS INFECTIONS |  |  |
| PARACOCCIDIOIDOMYCOSIS |  |  |
| PARAGONIMIASIS |  |  |
| PARAMYXOVIRIDAE INFECTIONS |  |  |
| PARAPARESIS, TROPICAL SPASTIC |  |  |
| PARASITE EGG COUNT |  |  |
| PARASITEMIA |  |  |
| PARASITIC DISEASES |  |  |
| PARASITIC DISEASES, ANIMAL |  |  |
| PARASITIC SENSITIVITY TESTS |  |  |
| PARATUBERCULOSIS |  |  |
| PARATYPHOID FEVER |  |  |
| PARONYCHIA |  |  |
| PAROTITIS |  |  |
| PARVOVIRIDAE INFECTIONS |  |  |
| PASTEURELLA INFECTIONS |  |  |
| PASTEURELLACEAE INFECTIONS |  |  |
| PASTEURELLOSIS, PNEUMONIC |  |  |
| PATIENT ISOLATION |  |  |
| PELVIC INFECTION |  |  |
| PELVIC INFLAMMATORY DISEASE |  |  |
| PENICILLIN RESISTANCE |  |  |
| PERICARDITIS, TUBERCULOUS |  |  |
| PERIMENINGEAL INFECTIONS |  |  |
| PERITONITIS |  |  |
| PERITONITIS, TUBERCULOUS |  |  |
| PERITONSILLAR ABSCESS |  |  |
| PESTE-DES-PETITS-RUMINANTS |  |  |
| PESTIVIRUS INFECTIONS |  |  |
| PHARYNGITIS |  |  |
| PHLEBOTOMUS FEVER |  |  |
| PICORNAVIRIDAE INFECTIONS |  |  |
| PIEDRA |  |  |
| PINTA |  |  |
| PISCIRICKETTSIACEAE INFECTIONS |  |  |
| PLAGUE |  |  |
| PLAQUE ASSAY |  |  |
| PLEURISY |  |  |
| PLEURODYNIA, EPIDEMIC |  |  |
| PLEUROPNEUMONIA |  |  |
| PLEUROPNEUMONIA, CONTAGIOUS |  |  |
| PNEUMOCOCCAL INFECTIONS |  |  |
| PNEUMOCYSTIS INFECTIONS |  |  |
| PNEUMONIA |  |  |
| PNEUMONIA OF CALVES, ENZOOTIC |  |  |
| PNEUMONIA OF SWINE, MYCOPLASMAL |  |  |
| PNEUMONIA, ASPIRATION |  |  |
| PNEUMONIA, ATYPICAL INTERSTITIAL, OF CATTLE |  |  |
| PNEUMONIA, BACTERIAL |  |  |
| PNEUMONIA, MYCOPLASMA |  |  |
| PNEUMONIA, PNEUMOCOCCAL |  |  |
| PNEUMONIA, PNEUMOCYSTIS |  |  |
| PNEUMONIA, PROGRESSIVE INTERSTITIAL, OF SHEEP |  |  |
| PNEUMONIA, RICKETTSIAL |  |  |
| PNEUMONIA, STAPHYLOCOCCAL |  |  |
| PNEUMONIA, VENTILATOR-ASSOCIATED |  |  |
| PNEUMONIA, VIRAL |  |  |
| PNEUMOVIRUS INFECTIONS |  |  |
| POLIOMYELITIS |  |  |
| POLIOMYELITIS, BULBAR |  |  |
| POLYOMAVIRUS INFECTIONS |  |  |
| POPULATION SURVEILLANCE |  |  |
| PORCINE POSTWEANING MULTISYSTEMIC WASTING SYNDROME |  |  |
| PORCINE REPRODUCTIVE AND RESPIRATORY SYNDROME |  |  |
| POUCHITIS |  |  |
| POULT ENTERITIS MORTALITY SYNDROME |  |  |
| POXVIRIDAE INFECTIONS |  |  |
| PREGNANCY COMPLICATIONS, INFECTIOUS |  |  |
| PREGNANCY COMPLICATIONS, PARASITIC |  |  |
| PRION DISEASES |  |  |
| PROSTHESIS-RELATED INFECTIONS |  |  |
| PROTEUS INFECTIONS |  |  |
| PROTOZOAN INFECTIONS |  |  |
| PROTOZOAN INFECTIONS, ANIMAL |  |  |
| PSEUDOMONAS INFECTIONS |  |  |
| PSEUDORABIES |  |  |
| PSITTACOSIS |  |  |
| PSOAS ABSCESS |  |  |
| PUERPERAL INFECTION |  |  |
| PULMONARY ASPERGILLOSIS |  |  |
| PYELITIS |  |  |
| PYELOCYSTITIS |  |  |
| PYELONEPHRITIS |  |  |
| PYOMETRA |  |  |
| PYOMYOSITIS |  |  |
| PYONEPHROSIS |  |  |
| PYURIA |  |  |
| Q FEVER |  |  |
| QUORUM SENSING |  |  |
| RABIES |  |  |
| RAT-BITE FEVER |  |  |
| RELAPSING FEVER |  |  |
| REOVIRIDAE INFECTIONS |  |  |
| RESPIRATORY PROTECTIVE DEVICES |  |  |
| RESPIRATORY SYNCYTIAL VIRUS INFECTIONS |  |  |
| RESPIRATORY TRACT INFECTIONS |  |  |
| RESPIROVIRUS INFECTIONS |  |  |
| RETICULOENDOTHELIOSIS, AVIAN |  |  |
| RETINITIS |  |  |
| RETROPHARYNGEAL ABSCESS |  |  |
| RETROVIRIDAE INFECTIONS |  |  |
| RHABDITIDA INFECTIONS |  |  |
| RHABDOVIRIDAE INFECTIONS |  |  |
| RHINOSPORIDIOSIS |  |  |
| RIBOTYPING |  |  |
| RICKETTSIA INFECTIONS |  |  |
| RICKETTSIACEAE INFECTIONS |  |  |
| RIFT VALLEY FEVER |  |  |
| RINDERPEST |  |  |
| RNA VIRUS INFECTIONS |  |  |
| ROCKY MOUNTAIN SPOTTED FEVER |  |  |
| ROSEOLOVIRUS INFECTIONS |  |  |
| ROTAVIRUS INFECTIONS |  |  |
| RUBELLA |  |  |
| RUBELLA SYNDROME, CONGENITAL |  |  |
| RUBIVIRUS INFECTIONS |  |  |
| RUBULAVIRUS INFECTIONS |  |  |
| SAFE SEX |  |  |
| SALMONELLA FOOD POISONING |  |  |
| SALMONELLA INFECTIONS |  |  |
| SALMONELLA INFECTIONS, ANIMAL |  |  |
| SARCOCYSTOSIS |  |  |
| SARCOMA, KAPOSI |  |  |
| SCABIES |  |  |
| SCHISTOSOMIASIS |  |  |
| SCHISTOSOMIASIS HAEMATOBIA |  |  |
| SCHISTOSOMIASIS JAPONICA |  |  |
| SCHISTOSOMIASIS MANSONI |  |  |
| SCRAPIE |  |  |
| SCREW WORM INFECTION |  |  |
| SCRUB TYPHUS |  |  |
| SECERNENTEA INFECTIONS |  |  |
| SENTINEL SURVEILLANCE |  |  |
| SEPSIS |  |  |
| SERIAL PASSAGE |  |  |
| SEROTYPING |  |  |
| SERRATIA INFECTIONS |  |  |
| SERUM BACTERICIDAL TEST |  |  |
| SETARIASIS |  |  |
| SEVERE ACUTE RESPIRATORY SYNDROME |  |  |
| SEXUALLY TRANSMITTED DISEASES |  |  |
| SEXUALLY TRANSMITTED DISEASES, BACTERIAL |  |  |
| SEXUALLY TRANSMITTED DISEASES, VIRAL |  |  |
| SHOCK, SEPTIC |  |  |
| SIALADENITIS |  |  |
| SILICOTUBERCULOSIS |  |  |
| SILVER STAINING |  |  |
| SIMIAN ACQUIRED IMMUNODEFICIENCY SYNDROME |  |  |
| SINUSITIS |  |  |
| SKIN DISEASES, BACTERIAL |  |  |
| SKIN DISEASES, INFECTIOUS |  |  |
| SKIN DISEASES, PARASITIC |  |  |
| SKIN DISEASES, VIRAL |  |  |
| SLOW VIRUS DISEASES |  |  |
| SMALL-AREA ANALYSIS |  |  |
| SMALLPOX |  |  |
| SOFT TISSUE INFECTIONS |  |  |
| SPACE-TIME CLUSTERING |  |  |
| SPARGANOSIS |  |  |
| SPECIFIC PATHOGEN-FREE ORGANISMS |  |  |
| SPHENOID SINUSITIS |  |  |
| SPIROCHAETALES INFECTIONS |  |  |
| SPIRURIDA INFECTIONS |  |  |
| SPOROTRICHOSIS |  |  |
| STAPHYLOCOCCAL FOOD POISONING |  |  |
| STAPHYLOCOCCAL INFECTIONS |  |  |
| STAPHYLOCOCCAL SCALDED SKIN SYNDROME |  |  |
| STAPHYLOCOCCAL SKIN INFECTIONS |  |  |
| STOMATITIS, HERPETIC |  |  |
| STREPTOCOCCAL INFECTIONS |  |  |
| STRONGYLE INFECTIONS, EQUINE |  |  |
| STRONGYLIDA INFECTIONS |  |  |
| STRONGYLOIDIASIS |  |  |
| SUBACUTE SCLEROSING PANENCEPHALITIS |  |  |
| SUBPHRENIC ABSCESS |  |  |
| SUPERINFECTION |  |  |
| SUPPURATION |  |  |
| SURGICAL WOUND INFECTION |  |  |
| SWINE ERYSIPELAS |  |  |
| SWINE VESICULAR DISEASE |  |  |
| SYPHILIS |  |  |
| SYPHILIS SERODIAGNOSIS |  |  |
| SYPHILIS, CARDIOVASCULAR |  |  |
| SYPHILIS, CONGENITAL |  |  |
| SYPHILIS, CUTANEOUS |  |  |
| SYPHILIS, LATENT |  |  |
| T-LYMPHOCYTOPENIA, IDIOPATHIC CD4-POSITIVE |  |  |
| TABES DORSALIS |  |  |
| TAENIASIS |  |  |
| TETANUS |  |  |
| TETRACYCLINE RESISTANCE |  |  |
| THEILERIASIS |  |  |
| THYROIDITIS, SUPPURATIVE |  |  |
| TICK INFESTATIONS |  |  |
| TICK-BORNE DISEASES |  |  |
| TINEA |  |  |
| TINEA CAPITIS |  |  |
| TINEA FAVOSA |  |  |
| TINEA PEDIS |  |  |
| TINEA VERSICOLOR |  |  |
| TOGAVIRIDAE INFECTIONS |  |  |
| TONSILLITIS |  |  |
| TOROVIRUS INFECTIONS |  |  |
| TOXASCARIASIS |  |  |
| TOXOCARIASIS |  |  |
| TOXOPLASMOSIS |  |  |
| TOXOPLASMOSIS, ANIMAL |  |  |
| TOXOPLASMOSIS, CEREBRAL |  |  |
| TOXOPLASMOSIS, CONGENITAL |  |  |
| TOXOPLASMOSIS, OCULAR |  |  |
| TRACHEITIS |  |  |
| TRACHOMA |  |  |
| TRANSFORMATION, BACTERIAL |  |  |
| TREMATODE INFECTIONS |  |  |
| TRENCH FEVER |  |  |
| TREPONEMA IMMOBILIZATION TEST |  |  |
| TREPONEMAL INFECTIONS |  |  |
| TRICHINELLOSIS |  |  |
| TRICHOMONAS INFECTIONS |  |  |
| TRICHOMONAS VAGINITIS |  |  |
| TRICHOSTRONGYLOIDIASIS |  |  |
| TRICHOSTRONGYLOSIS |  |  |
| TRICHURIASIS |  |  |
| TRIMETHOPRIM RESISTANCE |  |  |
| TROMBICULIASIS |  |  |
| TRYPANOSOMIASIS |  |  |
| TRYPANOSOMIASIS, AFRICAN |  |  |
| TRYPANOSOMIASIS, BOVINE |  |  |
| TUBERCULIN TEST |  |  |
| TUBERCULOMA |  |  |
| TUBERCULOMA, INTRACRANIAL |  |  |
| TUBERCULOSIS |  |  |
| TUBERCULOSIS, AVIAN |  |  |
| TUBERCULOSIS, BOVINE |  |  |
| TUBERCULOSIS, CARDIOVASCULAR |  |  |
| TUBERCULOSIS, CENTRAL NERVOUS SYSTEM |  |  |
| TUBERCULOSIS, CUTANEOUS |  |  |
| TUBERCULOSIS, ENDOCRINE |  |  |
| TUBERCULOSIS, FEMALE GENITAL |  |  |
| TUBERCULOSIS, GASTROINTESTINAL |  |  |
| TUBERCULOSIS, HEPATIC |  |  |
| TUBERCULOSIS, LARYNGEAL |  |  |
| TUBERCULOSIS, LYMPH NODE |  |  |
| TUBERCULOSIS, MALE GENITAL |  |  |
| TUBERCULOSIS, MENINGEAL |  |  |
| TUBERCULOSIS, MILIARY |  |  |
| TUBERCULOSIS, MULTIDRUG-RESISTANT |  |  |
| TUBERCULOSIS, OCULAR |  |  |
| TUBERCULOSIS, ORAL |  |  |
| TUBERCULOSIS, OSTEOARTICULAR |  |  |
| TUBERCULOSIS, PLEURAL |  |  |
| TUBERCULOSIS, PULMONARY |  |  |
| TUBERCULOSIS, RENAL |  |  |
| TUBERCULOSIS, SPINAL |  |  |
| TUBERCULOSIS, SPLENIC |  |  |
| TUBERCULOSIS, UROGENITAL |  |  |
| TULAREMIA |  |  |
| TUMOR VIRUS INFECTIONS |  |  |
| TYPHOID FEVER |  |  |
| TYPHUS, ENDEMIC FLEA-BORNE |  |  |
| TYPHUS, EPIDEMIC LOUSE-BORNE |  |  |
| UNSAFE SEX |  |  |
| UREAPLASMA INFECTIONS |  |  |
| URETHRITIS |  |  |
| URINARY TRACT INFECTIONS |  |  |
| UTERINE CERVICITIS |  |  |
| UVEITIS, SUPPURATIVE |  |  |
| VACCINATION |  |  |
| VACCINIA |  |  |
| VAGINOSIS, BACTERIAL |  |  |
| VANCOMYCIN RESISTANCE |  |  |
| VESICULAR EXANTHEMA OF SWINE |  |  |
| VESICULAR STOMATITIS |  |  |
| VIBRIO INFECTIONS |  |  |
| VIRAL INTERFERENCE |  |  |
| VIRAL LOAD |  |  |
| VIRAL TROPISM |  |  |
| VIREMIA |  |  |
| VIRULENCE |  |  |
| VIRUS ACTIVATION |  |  |
| VIRUS ASSEMBLY |  |  |
| VIRUS ATTACHMENT |  |  |
| VIRUS CULTIVATION |  |  |
| VIRUS DISEASES |  |  |
| VIRUS INACTIVATION |  |  |
| VIRUS INTEGRATION |  |  |
| VIRUS INTERNALIZATION |  |  |
| VIRUS LATENCY |  |  |
| VIRUS PHYSIOLOGICAL PHENOMENA |  |  |
| VIRUS PHYSIOLOGICAL PROCESSES |  |  |
| VIRUS RELEASE |  |  |
| VIRUS REPLICATION |  |  |
| VIRUS SHEDDING |  |  |
| VISNA |  |  |
| VULVOVAGINITIS |  |  |
| WARTS |  |  |
| WEIL DISEASE |  |  |
| WEST NILE FEVER |  |  |
| WHIPPLE DISEASE |  |  |
| WHOOPING COUGH |  |  |
| WOUND INFECTION |  |  |
| XENODIAGNOSIS |  |  |
| YAWS |  |  |
| YELLOW FEVER |  |  |
| YERSINIA INFECTIONS |  |  |
| YERSINIA PSEUDOTUBERCULOSIS INFECTIONS |  |  |
| ZOONOSES |  |  |
| ZOSTER SINE HERPETE |  |  |
| ZYGOMYCOSIS |  |  |
