# Supplementary material for: The State of Infectious Diseases Clinical Trials: A Systematic Review of ClinicalTrials.gov
Source: PLoS One. 2013 Oct 16;8(10):e77086. doi: 10.1371/journal.pone.0077086 (PMC3797691; doi:10.1371/journal.pone.0077086)
Supplement: Diagram S1 — PRISMA diagram. (DOC) [file pone.0077086.s006.doc]

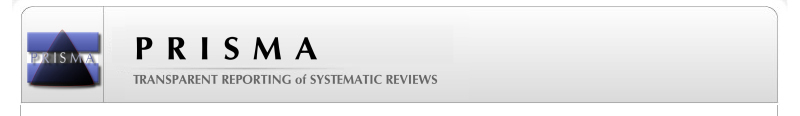
**Diagram S1. PRISMA 2009 Flow Diagram**

**Screening**

**Included**

**Eligibility**

**Identification**

Trials in CT.GOV database downloaded September 27, 2010 (n = 96 346)

Additional trials identified through other sources
(n = 0)

Trials after non-interventional and out of date range removed
(n = 40 970)

Trials screened using ID condition and intervention terms
(n = 40 970)

Trials excluded
(n =36 821)

Trials reviewed manually
(n = 4149)

Trials excluded

(n = 579)

Trials included in qualitative synthesis
(n = 3570)

Trials included in quantitative synthesis (meta-analysis)
(n = 3570 )
